# U2AF1 modulates alternative exon selection and guards zygotic splicing activation in mouse preimplantation embryogenesis

**DOI:** 10.1007/s00018-026-06197-y

**Published:** 2026-04-16

**Authors:** Zhi-Yan Jiang, Xuan Wu, Meng-Yan Jia, Lu Chen, Yu-Qi Liu, Heng-Yu Fan, Yin-Li Zhang

**Affiliations:** 1 MOE Key Laboratory for Biosystems Homeostasis and Protection and Innovation Center for Cell Signaling Network, Life Sciences Institute, Zhejiang University, Hangzhou, 310058 China; 2 Zhejiang Key Laboratory of Precise Protection and Promotion of Fertility, Department of Obstetrics and Gynecology, Sir Run Run Shaw Hospital, School of Medicine, Zhejiang University, Hangzhou, 310016 China; 3 State Key Laboratory of Female Fertility Promotion, Center for Reproductive Medicine, Department of Obstetrics and Gynecology, Peking University Third Hospital, Beijing, 100191 China

**Keywords:** Alternative splicing, Zygotic splicing activation (ZSA), Polypyrimidine tracts (PPTs), Female reproduction, Oocyte

## Abstract

Gene products vary in mRNA transcript and protein isoforms. During the maternal-to-zygotic transition, massive mRNAs transcribed from either the maternal or the zygotic genome were produced, together with a combination of pre-mRNA processing progress and mRNA decay mechanism. Transcripts harbored by embryos differ from the ones contained by oocytes in an alternative exon inclusion/exclusion manner. The lengths and the compositions of polypyrimidine tracts (PPTs) are crucial in splice site definition. Exons downwards longer PPTs were preferentially included, and less pyrimidine-incorporated PPTs tended to be excluded from 2-cell embryos. The U2AF dimers, U2AF1 and U2AF2, are responsible for PPT recognition and 3′ splice site determination. In oocytes, U2AF1 formed low density areas (cavities) and was positioned exclusively outside the nuclear speckles. In pre-implantation embryos, U2AF1 keeps in low abundance and disperses in the 2-cell nucleoplasm. Excessive U2AF1 expression leads to developmental arrest at the 2- to 4-cell stage, defective zygotic genome activation (ZGA), and aberrant zygotic splicing events. U2AF1 promotes exon exclusion in a PPT-dependent manner, modulates U2AF dimer’s recognition of more-pyrimidine-containing PPTs, defines 3′ splice sites, and selectively excludes the downstream exons. The composition of multiple gene variants, including *Bclaf1*, *Ezh2*, and several ZGA genes, is balanced by U2AF1 hypoexpression, which further assist in protein isoform maintenance, guarding proper protein positioning, and governing embryonic development.

**Supplementary Information:**

The online version contains supplementary material available at 10.1007/s00018-026-06197-y.

## Introduction

The RNA repertoire and a variety of protein products govern the viability, metabolism, and development of cells and organisms. Precursor mRNA (pre-mRNA) ought to be finely processed, including 5′ capping, 3′ cleavage, and polyadenylation, and splicing. Alternative splicing serves as a widespread mechanism, giving rise to diverse RNA variants and protein isoforms. Approximately 65% of murine and > 48% of human protein-coding genes express splicing variants [1–3]. Both co- and post-transcriptionally, spliceosomes together with various splicing regulators coordinate splice site recognition, intron branching, and exon ligation [4–6]. RNA-binding proteins that specifically interact with elements enriched in exons and introns are categorized as exonic and intronic splicing regulators, respectively. For example, high-purine motifs are predominantly bound by serine- and arginine-rich proteins (SR proteins) and promote exon skipping [7–9]. Such sequences are frequently observed in exons and are defined as exonic splicing enhancers. Other proteins, in combination with U1/2 small nuclear RNAs, are responsible for splice site recognition and determination.

During mammalian embryogenesis, a transition from the maternal to zygotic transcriptome occurs, which carries numerous nascent transcripts upon zygotic genome activation (ZGA). The accompanying processing machinery of zygotic splicing activation (ZSA) also emerges [10]. Owing to their diverse gene expression profiles and multiplex cassette usage, oocytes, zygotes, and blastomeres at different pre-implantation developmental stages contain diverse mRNA variants and protein isoforms [10–12]. Because of splicing strategies involving *Dnmt1*^exon1^, *Dnmt1* variants contain a downstream translation initiation site, thereby producing an N-terminal truncated active protein [13, 14]. Until the blastocyst stage, the oocyte-specific *Dnmt1* variant cannot be replaced by full-length *Dnmt1* mRNAs. When nuclear DPPA3 is removed from 2-cell embryos, an abbreviated, C-terminus-preserving form is restored in the cytoplasm and is associated with endosome recycling [15]. Proper splicing strategies and suitable protein isoforms are essential for both pre- and post-implantation embryogenesis [16]. Loss of the splicing machinery can result in embryonic lethality [17–19]. The blastomeres of mouse 2-cell embryos contain equal amounts of full-length *Dppa4* and its exon 4-excluded variant [10]. Excessive full-length DPPA4 results in 2- to 4-cell arrest in mouse embryos, whereas its isoform-depleted exon 4-coding peptides have minimal effect on development. Alternatively spliced exons of *Carm1* trigger multiple variants in mouse 2- and 4-cell embryos [20]. Dysregulation of splicing bias and its variant transition affects CARM1 protein abundance and cell fate determination. Oocytes also harbor cell type-specific variants. For instance, *Elavl2* encodes a maternal AU-rich element-binding protein. An ELAVL2 isoform resulting from exon skipping has been transiently detected in growing oocytes and is ablated upon meiotic maturation [21].

Several splicing regulators have been shown to facilitate maternal and zygotic pre-mRNA splicing. Splicing factors (i.e. SRSF1 [22], SRSF3 [23], RTCB [24]), and other protein factors (i.e. YTHDC1 [25], ESRP1 [26], and BCAS2 [27],) coordinate maternal mRNA processing. Spliceosome components, including *SNRPB* and *SNRPD2*, re required for U1/2/4/5/6 small nuclear ribonucleoprotein particle (snRNP) biogenesis and zygotic exon selection [12]. Inhibition of spliceosome activation dampens ZSA, leading to misprocessed transcripts and developmental arrest at the 2- to 4-cell stage [10]. Known as storage for pre-mRNA processing factors and transcripts, the presence of nuclear speckles significantly correlates with the splicing program. As placed at the central node of the nuclear speckles, SRRM2 and SON serve as scaffolds and independently regulate the splicing of mRNA target subsets in HeLa cells [28]. The depletion of *Son* in zygotes leads to massive alterations involving both exons and introns, defects in blastocyst formation, and transcriptomic disturbances [29]. Fine regulons are required to monitor and govern the splicing progress, obeying precise logic.

In this study, we compared alternative exon usage in oocytes and 2-cell embryos, and elucidated U2AF1 function in zygotic exon selection. Using an in vitro transcribed mRNA microinjection assay, we found that excessive U2AF1 impaired early embryogenesis and U2AF1 drove exon exclusion in a polypyrimidine tract (PPT)-dependent manner. Maintaining U2AF1 at low abundance at the 2-cell stage could be a prerequisite for ZSA; otherwise, a combination of mis-spliced transcripts, unsuitable mRNA variants, and inappropriate protein isoforms would be detrimental to embryonic development.

## Results

### Transcripts of oocyte and 2-cell embryo earn divert alternative exons

**Fig. 1:**
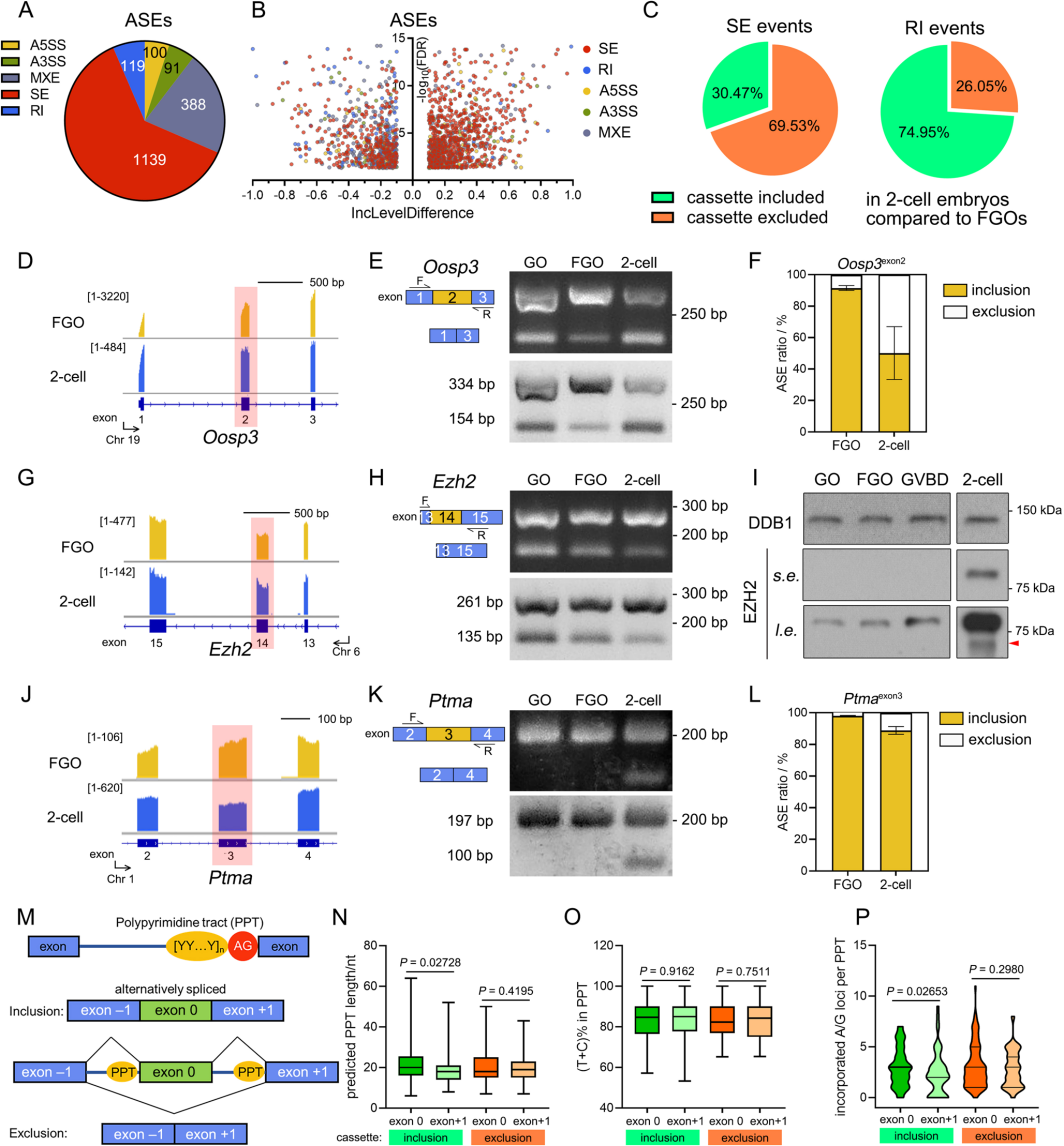
Comparison of alternative splicing events (ASEs) between wild-typed (WT) fully-grown oocytes (FGOs) and preimplantation mouse embryos at the 2-cell stage. **A**: Pie chart depicting the difference of ASEs between WT mouse FGOs and 2-cell embryos. Splicing events are labeled by indicated colors. Yellow, alternative-5′ spliced sites (A5SS). Green, alternative-3′ spliced sites (A3SS). Grey, mutually exclusive exons (MXE). Red, skipped exons (SEs). Blue, retained introns (RI). The amounts of the indicated ASEs are labeled within the sections, respectively. **B**: Volcano map of the inclusion (inc) level differences and false discovery rates (FDRs) of ASEs between the FGO and 2-cell transcriptomes. **C**: Pie charts showing the proportion of cassettes included and excluded in SE, and RI events comparing WT FGOs and 2-cell embryos. **D**: Schematic illustration by integrative genome viewer (IGV) analysis of mouse *Oosp3* isoforms in WT FGOs and 2-cell embryos. bp, base pair. **E**: PCR analysis of exon 2-containing *Oosp3* variants in growing oocytes (GOs), FGOs and 2-cell embryos. The localization of the primers is annotated. F, forward. R, reverse. **F**: Bar chart presenting *Oosp3*^exon2^ inclusion levels in transcriptome analysis. Mean ± SD. *n* = 3. **G-H**: IGV illustration and PCR analysis of *Ezh2* RNA variants in oocytes and 2-cell embryos. **I**: Western blotting results showing EZH2 protein isoforms in oocytes and embryos. 200 oocytes and embryos were loaded. DDB1 was applied as the loading control. *s.e.*, short exposure. *l.e.*, long exposure. **J-K**: IGV illustration and PCR analysis of *Ptma* RNA variants in oocytes and 2-cell embryos. **L**: Bar chart presenting the *Ptma*^exon3^ inclusion levels in transcriptome analysis. **M**: Schematic illustration of alternatively spliced exons. PPT, polypyrimidine tract. AG, adenine and guanine dinucleotide located at the intron-exon boundary, serving as the splicing acceptor site within the 3′-end of the intron. The alternative spliced exons are designated exon 0s (green boxes), and the corresponding upstream and downstream exons within the cassettes are designated exons –1 and +1, respectively (blue boxes). **N-O**: The lengths (**N**) and the proportion of thymines (Ts) and cytidines (Cs, **O**) within the predicted PPTs upstream the TOP 50 inclusive and exclusive cassettes according to the genome, respectively. nt, nucleotide. Central lines indicated the medians. Whiskers extended from min to max. **P**: Violin plot presented the incorporated purines loci within the predicted PPTs. Medians are indicated in the plots.

To investigate the differences between oocyte- and pre-implantation embryo-harboring transcripts, fully grown mouse oocytes (FGOs) and 2-cell embryos were subjected to SMART-seq2 sequencing and alternative splicing event (ASEs) analysis. The exon inclusion level (IncLevel) was measured to reflect the incorporation ratio of the indicated exons within isoforms and ranged from 0 to 1. The IncLevel difference (IncLevelDifference) of each exon was further applied to quantify the variation between FGOs and 2-cell embryos. Accordingly, ASEs could be categorized into 5 groups, including alternative 5′ and 3′ spliced sites (A5SS and A3SS, respectively), mutually exclusive exons (MXEs), skipped exons (SEs), and retained introns (RIs, Fig. S1A). 2-cell embryos harbored 1,837 ASEs when compared to FGOs, including 1,139 SEs (62.00%), 388 MXEs (21.12%), 119 RIs (6.49%), 100 A5SSs (5.44%), and 91 A3SSs (4.95%, Fig. 1A-B and Table S1). All ASE categories presented both cassette inclusion and exclusion, whereas more than a half of the alternative exons (69.53%) were excluded in 2-cell embryos, and 74.95% of alternative introns were retained (Fig. 1B-C and Fig. S1B).

Among them, well-known oocyte factors and genes essential for embryogenesis have been identified. An oocyte-secreted protein, OOSP3, genomically clusters with ovary-specific genes, was detected to be differentially spliced [30]. An alternative exon, *Oosp3*^exon2^, was more commonly included in FGOs than in 2-cell embryos (Fig. 1D-F). The lack of exon 2-coding peptides resulted in a frameshift, generating a premature termination codon and producing a short polypeptide containing only 27 amino acids (AAs, Fig. S1C). The catalytic subunit of polycomb repressive complex 2 (PRC2), EZH2, is responsible for zygotic H3K27 methylation [31, 32]. Within zygotes, EZH2 and its driven H3K27me3, known as repressive genome markers, are required for genome contraction [33]. Its alternative exon, *Ezh2*^exon14^, which is commonly differentially incorporated across different species, has been reported to be under shift-like regulation in pre-implantation embryos [12]. *Ezh2*^exon14^ presented a higher inclusion level in 2-cell embryos than in FGOs (Fig. 1G-H and Fig. S1D). Moreover, *Ezh2*^exon14^-incorporation preference was observed during the growth oocytes (from growing oocytes to FGOs), and the transition from oocytes to 2-cell embryos (Fig. 1H). The 126-base pair (bp)-long *Ezh2*^exon14^ encodes 42 amino acids (511-553AA, with one more AA transition from Asp to Gly), contributing to the Cys-X-Cys (CXC) domain (Fig. S1E). Loss of exon 14 could hamper the affinity of EZH2 for its substrate histone H3 tails as well as its cofactors JARID2 and AEBP2 [34, 35]. Although EZH2 protein levels were low in oocytes, a lower molecular weight EZH2 isoform was observed in 2-cell embryos, representing a much lower proportion compared to the full-length (fl) form (Fig. 1I). Other factors include diverted ASEs such as zygotic *Wee2* (also known as *Wee1b* and *Wee1* homolog 2) [36–39], of which the variants retained introns with an unannotated upstream translation initiation site (upTIS, Fig. S1F). Genes previously not annotated as essential in maternal-to-zygotic transitions also harbored diverse transcript variants at the 2-cell stage, *e.g. Ptma* (prothymosin alpha, involved in histone organization), selectively excluding exon 3 (Fig. 1J-L). Depletion of *Ptma*^exon3^-encoded glutamine-enriching peptides altered protein polarity, as annotated in Uniprot (Fig. S1G).

Various exonic and intronic elements, as well as multiple RNA-recognizing factors are involved in exon selection. At the 3′ splice sites around intron-exon junctions, a group of splicing signals exist: a branchpoint sequence (BPS), a PPT, and an invariant AG dinucleotide adjacent to the downstream exon (Fig. 1M) [40]. The existence of PPTs within introns was required for spliceosome assembly and accurate selection of exons [41, 42] A longer sequence, a high pyrimidine incorporation ratio, and appropriate localization would strengthen the role of PPT to splicing decisions. To decipher whether PPTs contribute to 2-cell embryo exon alternation, SEs of the TOP 50 inclusive and exclusives cassettes were further investigated according to IncLevelDifference (Table S2). Here, SE itself was indicated as exon 0, and its downstream exon (exon +1) was analyzed (Fig. 1M). As of inclusive exon 0s in 2-cell embryos, their upstream PPTs were slightly longer than those of exon + 1 (median 18 to 20 nt, Fig. 1N). The corresponding pyrimidine ratios were similar for both PPTs, with averages of 84.61% and 85.00% (Fig. 1O). Predicted PPTs upstream, exclusive exon 0s at the 2-cell stage, were more similar to their downstream counterparts, with median PPT lengths of 18 and 19 nt, and higher pyrimidine ratios averaging 82.35% and 84.21%, respectively. In addition, introduction of purines into PPTs is detrimental to splicing [42]. PPTs upstream of both included or excluded exon 0s in 2-cell embryos were more frequently segmented by purines, and they would be localized relatively farther from the AG dinucleotides (Fig. 1P and Fig. S1H). This indicates that PPT properties could affect downstream exon selection, especially in 2-cell embryo included exons, and contribute to diverting transcript variants in oocytes and embryos.

### PPT-recognizing U2AFs are expressed in mouse oocytes

**Fig. 2:**
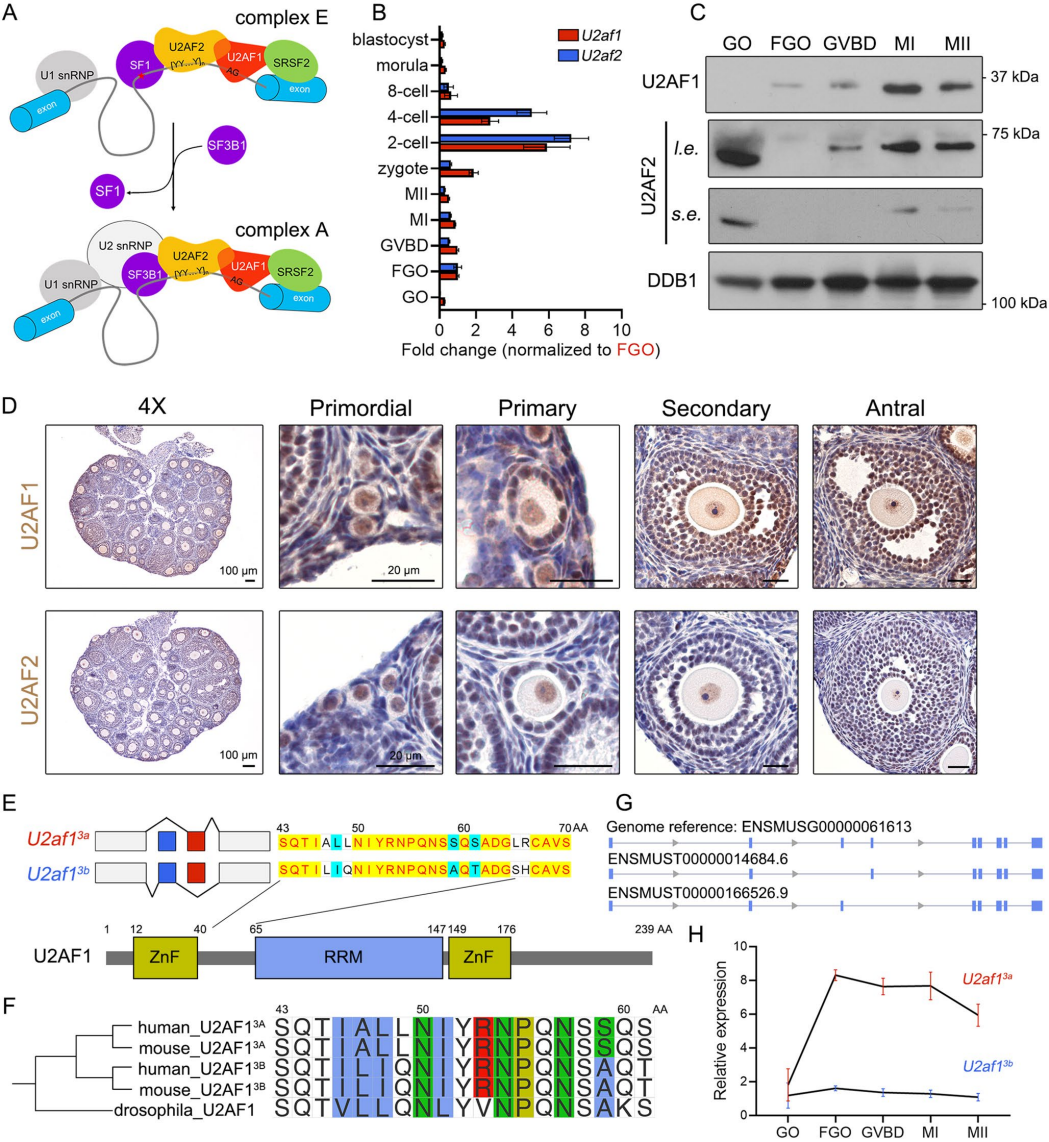
Expression patterns of two U2 snRNP co-factors, U2AF1/2, in mouse oocytes during folliculogenesis and meiotic maturation. **A**: Schematic diagram depicting U2AF1/2 assembly in complexes E and A. U2AF2 recognizes the PPTs (designated as [YY…Y]_n_) and U2AF1 binds to the downstream AG dinucleotide repeats. **B**: qRT-PCR results showing the relative expression levels of *U2af1/2* transcripts in mouse oocytes and preimplantation embryos at the indicated stages. Mean ± SEM. *n* = 3. **C**: Western blotting results showing U2AF1/2 protein abundance in oocytes undergoing meiosis. DDB1 served as the loading control. **D**: IHC staining presenting the expression of U2AF1/2 in mouse ovaries and follicles at the indicated stages. Scale bars, 100 μm in 4× microscopy and 20 μm in enlarged views. **E**: Comparison between the two MXEs, exons 3a/3b in mouse *U2af1* and their coding peptides. AA, amino acid. **F**: Comparison of U2AF1 exon 3-coding peptides among species. **G**: Schematic illustration of mouse *U2af1* genomic DNA, and exon 3a/b-containing transcripts. **H**: qRT-PCR results showing the relative abundance of *U2af1*^*3a/3b*^ mRNA variants in oocytes during meiotic maturation. Mean ± SEM. *n* = 3

The spliceosome is a multiprotein complex that assembles in a highly ordered manner and undergoes a series of conformational changes during pre-mRNA processing [6]. Early spliceosome (E-complex) formation is initiated upon U1 snRNPs loading to 5′ splice sites, whereas at the 3′-end, splicing factor 1 (SF1) anchors the BPS, and U2 small nuclear RNA auxiliary factors (U2AFs) target the PPT (Fig. 2A). Transformation from the E- to A-complex (also known as the pre-spliceosome) occurs as SF3B1 substitutes for SF1 at the branch helix, followed by U2 snRNP recruitment by U2AFs. U2AF homologs, namely U2AF1 (also known as U2AF35) and U2AF2 (also known as U2AF65), form heterodimers and coordinate in pre-mRNA 3′ splice site processing. As U2AF1 recognizes AG dinucleotides ahead of exons, U2AF2 contacts PPTs. To determine whether U2AFs are required for oocyte and embryonic exon selection, we first evaluated *U2af1/2* expression in mouse tissues and germ cells (Fig. S2A). Abundant in the reproductive system, *U2af1/2* was expressed in oocytes during folliculogenesis and meiotic maturation, and in pre-implantation embryos (Fig. 2B and Fig. S2B). Oocytic *U2af1/2* mRNA levels were decreased after germinal vesicle breakdown (GVBD), indicating that they represent oocyte decay factors (Fig. S2C). Western blotting results indicated that U2AF1/2 underwent translational activation accompanied by meiotic resumption, probably serving as a preparatory reserve for early embryogenesis (Fig. 2C). Although at a lower abundance, U2AF1/2 proteins were expressed in oocytes at all follicular stages (Fig. 2D). Both proteins were localized in the nucleus, whereas U2AF2 were highly expressed in oocytes comparing to ovarian somatic cells, and U2AF1 was also expressed in granulosa cells.

The *U2af1* gene itself can be alternatively spliced and contains a pair of MXEs, namely, exons 3a and 3b [43]. Both exons 3a/b are 67 nt in length and encode a peptide linking the C-terminal zinc finger (ZnF) domain and the RNA-recognizing motif (RRM, also known as the U2AF homology motif (UHM)), with only seven AAs in difference, and maintain interaction with U2AF2 (indicated as U2AF^3A^ and U2AF^3B^, respectively, Fig. 2E-F). *U2af1* transcripts harboring both exon 3a and 3b are cleared owing to a premature termination codon incorporation [43]. The coexistence of U2AF^3A^ and U2AF^3B^ was evolutionarily conserved, as various mammalian U2AF1 isoforms were present, whereas *Drosophila* U2AF1 had only one form (Fig. 2G). Previous studies have reported that U2AF^3A/B^ exhibits heterogeneity in substrate sequence preferences [44]. However, U2AF^3A^ plays a decisive role in HEK293 cells due to its high abundance [45]. Real-time quantitative PCR (qRT-PCR) results also revealed the predominant constitution of *U2af1*^*3a*^, with *U2af1*^*3b*^ at the basic expression level (Fig. 2H).

### U2AF1 forms cavities exclusive to nuclear speckles in mouse oocytes

**Fig. 3:**
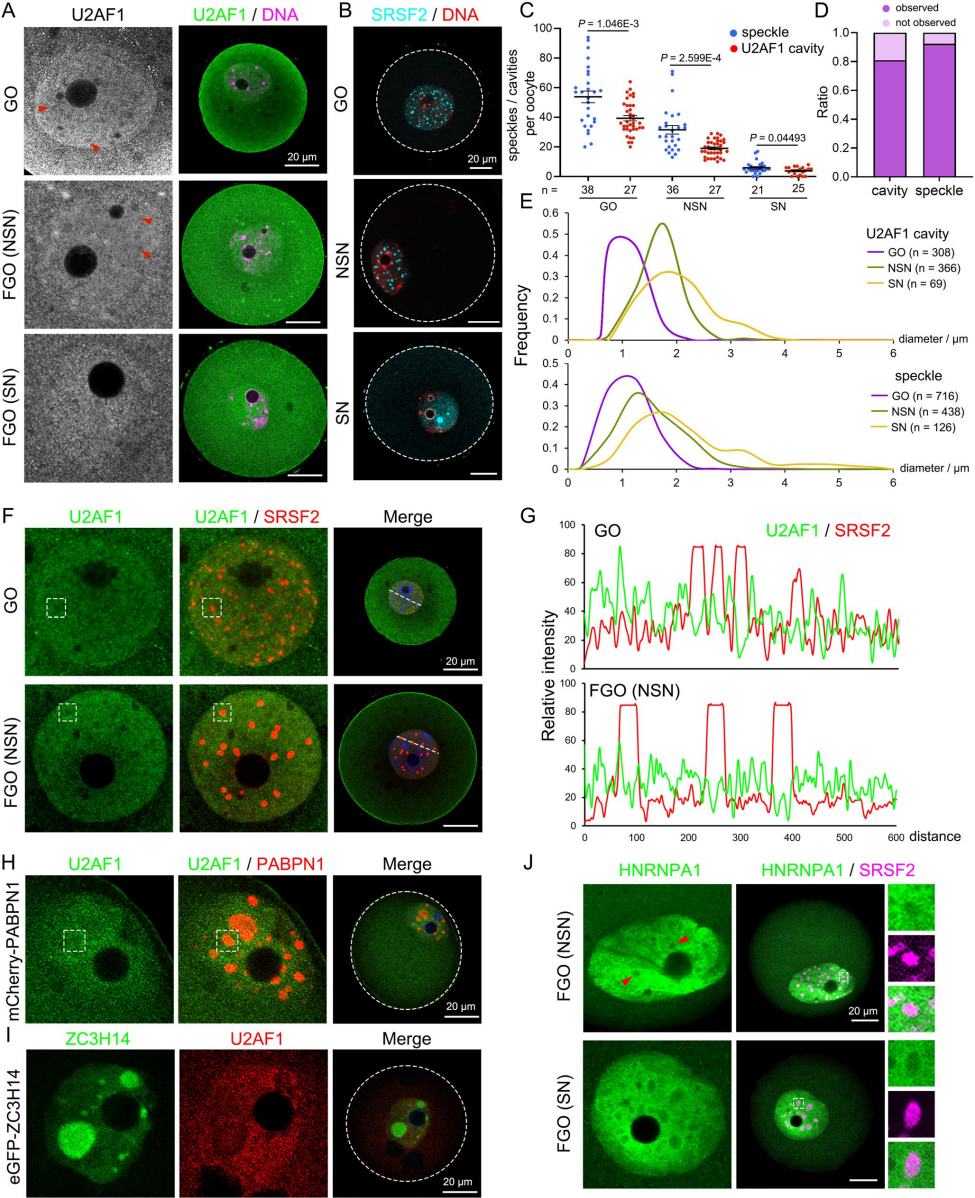
U2AF1 distributes exclusively outside nuclear speckles in mouse oocytes. **A-B**: Immunofluorescent (IF) staining of U2AF1 (**A**) and nuclear speckles (indicated by SRSF2, **B**) in mouse oocytes. Red triangles point at U2AF1 cavities. Scale bars, 20 μm. **C**: The amounts of U2AF1 cavities and nuclear speckles per oocyte. Mean ± SEM. Numbers of the analyzed oocytes (n) are annotated at the bottom. **D**: Bars presenting the proportions of SN-stage FGOs observed with or without cavities/nuclear speckles. **E**: Comparison of the cavity/nuclear speckle diameters in oocytes at the indicated stages. Numbers of the analyzed oocytes (n) are annotated on the right. **F-G**: IF staining of U2AF1 and SRSF2 in GOs and NSN-stage oocytes. The distribution of U2AF1 and SRSF2 (**G**) was analyzed along the white dashed lines using ImageJ. Scale bars, 20 μm. **H-I**: IF staining of U2AF1 and ectopically expressed mCherry-PABPN1 and eGFP-ZC3H14 in mouse oocytes. Scale bars, 20 μm. **J**: IF staining of HNRNPA1 and SRSF2 in NSN- and SN-stage oocytes. Red triangles point at HNRNPA1 cavities. Scale bars, 20 μm

Nuclear speckles, which are membraneless bodies within the nucleoplasm, are known as hubs for pre-mRNA splicing. A group of mRNA processing factors, including certain snRNPs, splicing factors (e.g. SR proteins), and cleavage and polyadenylation proteins, are engaged in the speckles [28, 46–48]. Relying on their mixed-charge domains and RRMs that interact with RNA substrates, these proteins condense and are incorporated into nuclear speckles [49]. Resembling somatic cells, various splicing partners have been demonstrated to be nuclear speckle components in oocytes, including SRSF2 [50], PABPN1 [51], UBE2I [52] and BCAS2 [27]. To assess whether U2AFs could also assemble into nuclear speckles, GOs and FGOs were collected and stained using anti-U2AF1 antibodies. Surprisingly, U2AF1 was unevenly distributed in the nucleoplasm, forming multiple low-density areas detected by immunofluorescence staining (U2AF1 cavities; Fig. 3A). These cavities were prevalent in GOs, and decreased in non-surrounded nuclear (NSN)-stage FGOs, whereas U2AF1 was more evenly distributed in SN-stage FGOs (Fig. 3A). This pattern was similar to that observed in the nuclear speckles (Fig. 3B). GOs contained a large number of speckles (54 ± 21 per oocytes) and U2AF1 cavities (39 ± 11 per oocytes), which were small in size, with average diameters of 1.147 ± 0.3787 and 1.081 ± 0.2735 μm, respectively (Fig. 3C and Fig. S2D-F). As for NSN-stage FGOs, the amounts of speckles and U2AF1 cavities were decreased (32 ± 15, and 19 ± 6 per oocyte, respectively), and enlarged in volume (with average diameters of 1.561 ± 0.6103, and 1.666 ± 0.3811 μm). Both U2AF1 cavities and speckles were less common in SN-staged oocytes (detected in 80.95% and 92.31% of the analyzed oocytes, respectively; Fig. 3D). Regardless of a few larger speckles, U2AF1 cavities, sharing similar sizes to speckles, were observed in same-stage oocytes (Fig. 3E).

To verify the localization of U2AF1 and nuclear speckles, SRSF2, a well-studied nuclear speckle marker, was co-stained with U2AF1. Interestingly, SRSF2 was positioned exactly in low-U2AF1-density areas (Fig. 3F-G). Other speckle components, including PABPN1 and ZC3H14, were also distributed exclusively in U2AF1 oocytes (Fig. 3H-I and Fig. S2G). Other *trans* factors that primarily recognize intronic elements, such as HNRNPA1, have been reported to be depleted or not enriched in speckles [53, 54]. In oocytes, HNRNPA1 also formed cavities occupied by SRSF2-indicated speckles (Fig. 3J and Fig. S2H). These results clarified that U2AF1 was depleted in the nuclear speckles of mouse oocytes, suggesting that the boundaries of speckles may be important in pre-mRNA processing.

### U2AF2 assemblies into nuclear speckles only in surrounded nuclei staged oocytes

Unlike the U2AF1 positioning pattern, U2AF2 was evenly distributed in the nucleoplasm with few high-density puncta in NSN-stage oocytes (Fig. S3A). In SN-staged oocytes, U2AF2 was assembled in larger spots, excluding the chromatin. U2AF2 can form liquid-like condensates together with its bound PPTs, and is enhanced by SF3B1 and other cofactors [55]. Condensed U2AF2 was also observed in follicular oocytesin situ, but only in large antral follicles, by immunofluorescence and immunohistochemical staining (Fig. S3B-C). Co-staining for SRSF2 and U2AF2 clarified that U2AF2 was indeed incorporated into speckles in SN-stage oocytes (Fig. S3D-E). In GOs and NSN-stage oocytes, U2AF2 exhibited no spatial preference for regions within, adjacent to, or external to nuclear speckles. The assembly of U2AF2 into speckles did not correspond to speckle volumes; U2AF2 was evenly localized around speckles in GOs. In SN-stage oocytes, U2AF2 was enriched in both larger speckles and the ones similar to those in GOs in size (Fig. S3F). Collectively, U2AF2 acts as a stage-specific nuclear speckle passenger and the divergent positioning of U2AF1/2 implies functional differences during development.

### U2AF1 hyperexpression leads to ZGA failure and developmental arrest at 2-cell stage

**Fig. 4:**
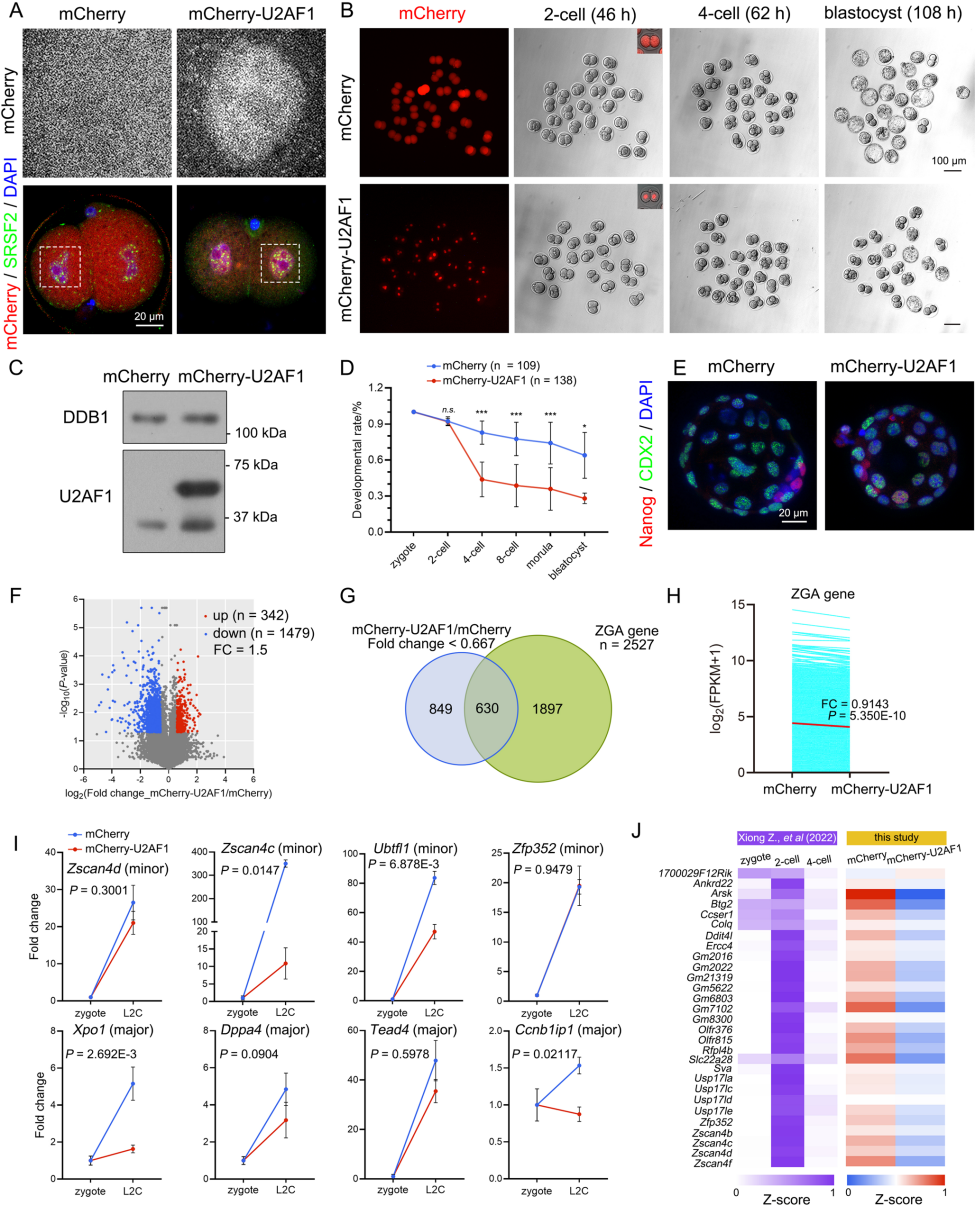
Ectopically expressed U2AF1 resulted in embryonic development arrest at the 2- to 4-cell stage. **A**: IF staining of SRSF2 and microinjected mCherry-U2AF1 in 2-cell embryos. Scale bars, 20 μm. **B**: Representative images of mouse embryos microinjected with *mCherry* or *mCherry-U2AF1* mRNAs at the indicated stages. The observation time points post human chronic gonadotropin (hCG) are labeled. Scale bars, 100 μm. **C**: Western blotting results showing U2AF1 overexpression in microinjected groups. DDB1 was applied as the loading control. **D**: Developmental rates of U2AF1 ectopically expressed embryos at the indicated stages. Embryos subjected to *mCherry* mRNA microinjection were applied as the control. Mean ± SD. Numbers of the analyzed embryos (n) are annotated on the top. **E**: IF staining of CDX2 and Nanog in the blastocysts and the indicated groups. Scale bars, 20 μm. **F**: Volcano map of the differently expressed genes (DEGs) at the mRNA level between mCherry-U2AF1- and mCherry-expressed 2-cell embryos. Upregulated transcripts were defined as the ones whose fold change (FC) over 1.5 in FPKM_mCherry−U2AF1_/FPKM_mCherry_ and *P* < 0.05, indicated as red dots. Downregulated transcripts are colored in blue, vice versa. **G**: Venn diagram comparing the downregulated genes in U2AF1 ectopically expressed 2-cells and commonly defined zygotic genome activation (ZGA) genes. **H**: Expression of ZGA genes in mCherry and mCherry-U2AF1 expressed 2-cell embryos. The red line shows the average level of both groups. **I**: qRT-PCR results showing the expression levels of ZGA gene transcripts in embryos from the indicated groups. Mean ± SEM. *n* = 3. **J**: Heatmaps presenting the expression of representative totipotent gene transcripts in WT embryos (left, referred to Xiong Z., et al.) and U2AF1 hyperexpression 2-cell embryos (right)

Maternal products, including organelles, proteins, and mRNAs, are stored in oocytes and guard fertilization and early embryogenesis. In addition to the transcription protram, newly transcribed pre-mRNAs must be processed, which is referred to as the ZSA program. As oocytic nuclear envelope breakdown, chromatin condensation, and nuclear speckle components are dispersed into the cytoplasm. At approximately 18 h post fertilization, nuclear speckles reformes in 2-cell nuclei, accompanied by vigorous transcription [56, 57]. Although U2AF1 was translationally activated upon GVBD, U2AF1 was low in abundance, and only basic expression was detected in the 2-cell embryo nucleoplasm (Fig. S4A-B). Thus, U2AF1 cavities were not detected. To address whether the exclusive distribution of U2AF1 and speckles was maintained in pre-implantation embryos, in vitro transcribed *mCherry-U2AF1* mRNA was microinjected into zygotes. Since U2AF1^3A^ served as the predominant form among cell types and across species, *U2AF1*^3A^ was cloned and subjected to further analyses. Although ectopic U2AF1 resided in the nucleus, U2AF1 was evenly distributed within the nucleoplasm, disregarding nuclear speckle positioning (Fig. 4A). Moreover, U2AF1 hyperexpression resulted in developmental arrest at the 2- to 4-cell stage (Fig. 4B-D). Only 28.05% of U2AF1-hyperexpressing cells developed into blastocysts, which were smaller in size and contained fewer blastomeres (Fig. 4E).

To elucidate the effect of hyperexpressed U2AF1 on 2-cell transcriptomes, late 2-cell embryos ectopically expressing mCherry and mCherry-U2AF1 were subjected to SMART-seq2 analysis. Gene expression levels were assessed as fragments per kilobase of transcript per million mapped reads (FPKM). ERCC spike-in was applied for gene expression correction (Table S3, Fig. S4C-D). A total of 342 and 1479 genes were upregulated and downregulated in the U2AF1 hyperexpression group, respectively, compared to those in the mCherry group (fold change = 1.5, *P*-value < 0.05; Fig. 4F). Among these, 42.60% of downregulated genes were ZGA genes [58, 59] (Fig. 4G). As ZGA gene expression exhibited a global reduction, most of them were activated, but to a lower extent than in the control group (Fig. 4H-I). A small group of these genes, including *Ccnb1ip1*, evidenced decreased mRNA levels under excessive U2AF1 conditions. An overwhelming decrease in totipotent gene expression was observed in the U2AF1 hyperexpression group, whereas pluripotent gene products were transcribed in a disordered manner (Fig. 4J and Fig. S4E). Taken together, hyperexpressed U2AF1 results in defects in pre-implantation embryogenesis, leading to ZGA failure and developmental arrest at the 2- to 4-cell stage.

### ZSA resulting from U2AF1 hyperexpression is accompanied by exon selection downstream of high-pyrimidine PPTs

**Fig. 5:**
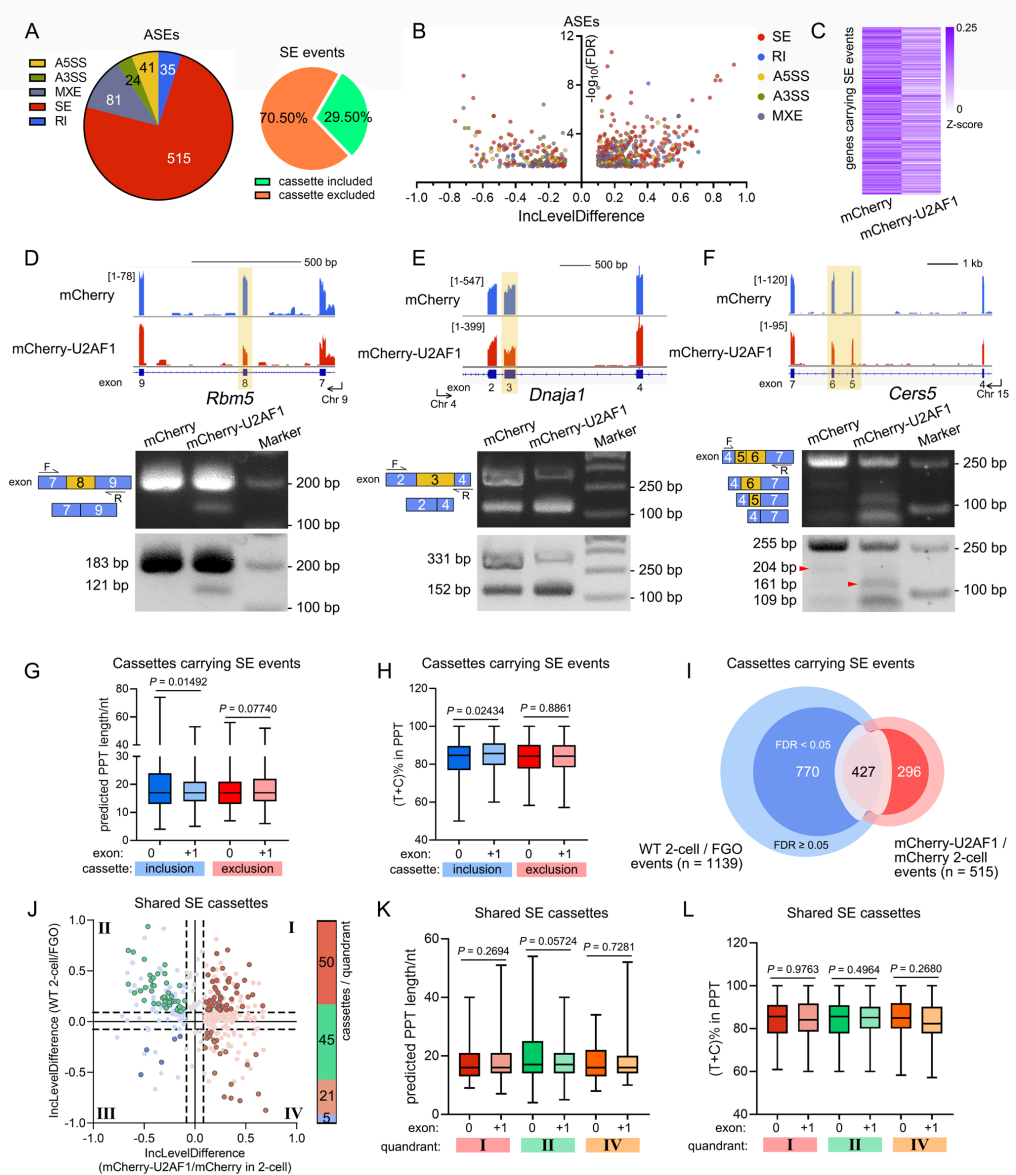
Selective exon exclusion in 2-cell embryos upon excessive U2AF1. **A**: Pie chart depicting the difference of ASEs in 2-cell embryos ectopically expressing mCherry-U2AF1 or mCherry. Splicing events are labeled by indicated colors. The cassettes either being included or excluded in SE events are presented on the right. **B**: Volcano map of the inclusion level differences and FDRs of ASEs between the ectopic mCherry-U2AF1- and mCherry-expressing 2-cell transcriptomes. **C**: Heatmap presenting the mRNA abundance of genes harboring different SEs in the corresponding groups. **D-F**: Schematic illustration by IGV and PCR analyses of *Rbm5*,* Dnaja1*, and *Cers5* variants in mCherry- and mCherry-U2AF1-expressing 2-cells. Red triangles in **F** indicated the exon 5/6-containing *Cers5* variants. **G-H**: The lengths (**G**) and the proportion of pyrimidines (**H**) within the predicted PPTs upstream the alternative exons upon U2AF1 hyperexpression in 2-cells, respectively. Central lines indicated the medians. Whiskers extended from min to max. *Hmgn5*^exon2^ was not shown due to derivation. **I**: Venn chart showing the cassettes carrying SE events in U2AF1-ectopic expressing 2-cells, compared to the ones differed in FGOs and WT 2-cell embryos. Cassettes presented significant difference either upon U2AF1 hyperexpression or in 2-cells compared to FGOs are highlighted and further investigated. Numbers of ASEs (n) detected in the comparison of WT 2-cell/FGO and mCherry-U2AF1/mCherry-expressing 2-cells are annotated. Cassettes with higher fidelity (FDR < 0.05) are placed in the inner circle, while the rest (FDR ≥ 0.05) are colored in light colors. **J**: Scatter plot comparing the IncLevelDifferences of SE-carrying cassettes during FGO-to-2-cell transition and in U2AF1 hyper- or hypo-expressing 2-cells. The amounts of cassettes with high fidelity are indicated on the right. **K-L**: Box plots showing the predicted PPT lengths (**K**), and the pyrimidine ratios (**L**) upstream the exons in the corresponding quadrants in **J**. Central lines indicated the medians. Whiskers extended from min to max

Considering the primary function of U2AF1 in pre-mRNA processing, splicing alterations were analyzed using rMATS [60], comparing ectopic U2AF1-expressing and control groups (Table S4). U2AF1 hyperexpression resulted in 81 MXEs (11.64%), 35 RIs (5.03%), 41 A5SSs (5.89%), and 24 A3SSs (3.45%, Fig. 5A-B). As the majority of the ASEs, excessive U2AF1 resulted in 515 SE events. 70.50% of the SE cassettes were excluded in 2-cell embryos ectopically expressing U2AF1. These SE-carrying genes also exhibited reduced abundance in transcriptome analysis (Fig. 5C). However, the overall expression levels of the splicing factors remained similar with or without U2AF1 microinjection (Fig. S5A). While a small group of maternal splicing factors (*i.e. Srsf3*) was downregulated in U2AF1-hyperexpressing 2-cell embryos, the transcriptional abundance of most zygotic and general splicing factors, including *Sf3b1*,* Srsf2*,* Son*, and *Srrm2*, remained stable compared to that in the control cohort (Fig. S5B-C).

We examined the U2AF1-implied exon selection for SEs. Among the 515 SE events, 341 gene products presented with exon alternations. For instance, *Rbm5*^exon8^ and *Dnaja1*^exon3^ were preferentially excluded in 2-cell embryos upon U2AF1 overexpression (Fig. 5D-E and Fig. S5D). For *Cers5*, a variant containing exon 4/5/7 was selectively assembled (Fig. 5F). In the control group, only full-length *Cers5* and a small proportion of exons 4/6/7-containing variants were detected. All three genes were downregulated in U2AF1-hyperexpressing 2-cell embryos (Fig. S5E). PPTs along the differentially incorporated exons under U2AF1 hyperexpression conditions varied in length, from 5 nt ahead *Creld2*^exon3^ to 95 nt upstream *Hmgn5*^exon2^ (Table S5). Regardless of the preference for inclusion or exclusion, PPTs upstream of the U2AF1-altered exon 0s showed similar lengths to their exon +1s (17 at the median of all four groups, Fig. 5G). Comparable pyrimidine ratios were detected in U2AF1-induced excluded exon 0s, with average levels of 83.91% and 83.99% of the corresponding exon +1s (Fig. 5H). PPTs of the inclusion cassettes incorporated less pyrimidine residues upstream of exon 0s, averaging 83.22% of exon 0s and 85.20% of exon +1s. Although the predicted PPTs upstream of *Rbm5*^exon8^, *Dnaja1*^exon3^, and *Cers5* ^exon5/6^ were relatively shorter than those upstream of the corresponding exon +1s, higher pyrimidine ratios existed (Fig. S5F). These results imply that U2AF1 is induced in the exon 0 exclusion downstream of higher T/C-containing PPTs.

By integrating the SE events between wild-type 2-cell embryos to FGOs, and ectopic U2AF1 expression to the control 2-cell group, we further investigated whether cassettes commonly underwent exon alternation. SE cassettes presented significant differences (IncLevelDifference > 0.1 or < −0.1), either in FGO-to-2-cell transition or upon U2AF1 hyperexpression in the 2-cell embryos analyzed (Fig. 5I). In this study, 286 genes harboring 427 SE cassettes were identified. Among these, 268 and 135 exons were excluded and included, respectively, in ectopic U2AF1-expressing 2-cell embryos among the shared cassettes (quadrants Ⅰ and Ⅳ for exclusion and quadrants Ⅱ and Ⅲ for inclusion, Fig. 5J). For 2-cell selectively excluded exons with high fidelity (false discovery rate (FDR) < 0.05), a balanced inclusion/exclusion ratio was observed upon U2AF1 introduction (45 and 50 cassettes, accounting for 41.32% and 37.19%, respectively). Only five cassettes were both inclusive in 2-cells and in the U2AF1-driven group (quadrant Ⅲ), which were not further investigated. The lengths of the predicted PPTs upstream cassettes were similar in all three quadrants, with medians of exon 0s and +1s at 16 in quadrants Ⅰ and Ⅳ, and 17 in quadrant Ⅱ (Fig. 5K). Although PPTs of the cassettes in quadrant Ⅱ harbored the highest T/C ratios (85.71% and 85.19% of exon 0s and exon +1s, respectively), cassettes within quadrants Ⅰ and Ⅳ showed higher pyrimidine incorporation in PPTs ahead of exon 0s than those upstream exon +1s (83.77% and 82.35% in quadrant Ⅰ, and 84.95% and 82.35% in quadrant Ⅳ, Fig. 5L). Collectively, U2AF1 modulates exon integration during zygotic mRNA processing in 2-cell embryos, prefers exons downstream of high-pyrimidine PPTs, and mainly induces exclusion cassettes that would be removed during ZSA. Disorderly spliced transcripts are cleared by RNA surveillance and other mRNA decay machinery, resulting in lower gene expression levels and hampering embryogenesis.

### U2AF1-modulated *Bclaf1*^exon11^ exclusion changes BCLAF1 distribution and impedes pre-implantation embryogenesis

**Fig. 6:**
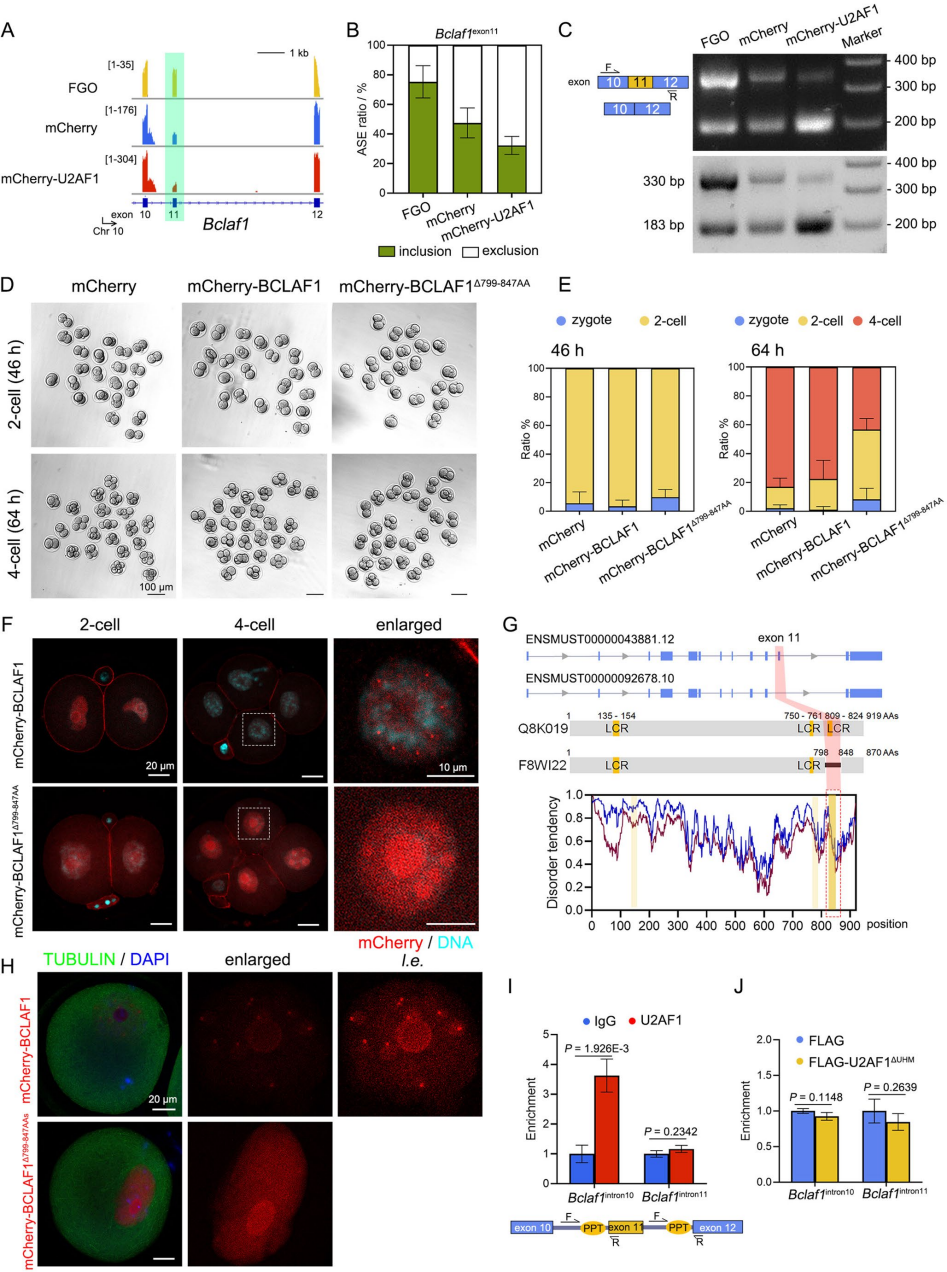
BCLAF1 isoform lacking of exon 11-coding peptides resulted in mouse embryonic development arrest at the 2-cell stage. **A**: IGV illustration of *Bclaf1* transcripts in WT FGO, mCherry- and mCherry-U2AF1-expressing 2-cell transcriptomes. The genome reference is presented at the bottom. **B**: Ratios of *Bclaf1* transcripts including or excluding exon 11 in the FGOs, mCherry- and mCherry-U2AF1-expressing 2-cells. Mean ± SEM. *n* = 3. **C**: PCR analysis of *Bclaf1* RNA isoforms in the indicated groups. **D**: Representative images of mouse embryos microinjected with *mCherry*, *mCherry-Bclaf1*, and *mCherry-Bclaf1*^Δexon11^ mRNAs (labeled as mCherry-Bclaf1^Δ799–847AA^) at the indicated time point. Scale bars, 100 μm. **E**: Developmental rates of the indicated groups at 46 h (left) and 64 h (right) post hCG treatment. Mean ± SEM. **F**: Confocal microscopy of mCherry-BCLAF1 isoforms in 2-cell and 4-cell embryos. Scale bars, 20 μm. **G**: Schematic illustration of *Bclaf1* variants either containing exon 11 or not (top) and disorder analysis of BCLAF1 protein (bottom). LCR, low complexity region colored in yellow. The exon 11-encoding peptide is indicated in red. **H**: IF results showing TUBULIN and BCLAF1 variants in FGOs. Scale bars, 20 μm. **I-J**: qRT-PCR results showing the affinity between endogenous U2AF1 (**I**) and UHM-lacking U2AF1 (**J**) to *Bclaf1* mRNA regions analyzing by RNA-immunoprecipitation (RIP). The enrichment of the indicated transcript regions was normalized by the IgG and FLAG-expressing group, respectively. Mean ± SEM. *n* = 3. The design of primers is presented at the bottom

A typical example of U2AF1-induced, 2-cell preferred exon exclusion was carried out involving *Bclaf1* (Fig. 6A-C and Fig. S6A). In the human placenta, *BCLAF1* serves as a random allelic gene that preferentially expresses maternal alleles [61]. As a multiple exon-coding gene, *BCLAF1* contains two alternative exons: exon 5a for A5SS and exon 11 for SE [62]. In human and mouse tissues, *BCLAF1*^exon11^ is commonly included. In mouse oocytes, *Bclaf1*^exon11^ was also overwhelmingly included (Fig. 6A and Fig. S6A). We thus cloned *Bclaf1*, constructed the exon 11-deleted variant (annotated as BCLAF1^Δ799–847AA^), and examined its effects on embryogenesis. Embryos ectopically expressing mCherry-tagged BCLAF1 developed normally into blastocysts, whereas introduction of BCLAF1^Δ799–847AA^ resulted in developmental arrest at the 2- to 4-cell stage (Fig. 6D-E). Both BCLAF1 isoforms were mainly localized in the nucleus and dispersed in the nucleoplasm (Fig. 6F). In 4-cell embryos, however, BCLAF1^Δ799–847AA^ accumulated inside the nucleoli, whereas full-length BCLAF1 aggregated into puncta and became faint in the nucleoplasm. Similarly, failure in puncta formation was also observed in ectopically BCLAF1^Δ799–847AA^-expressing FGOs (Fig. 6H). As the 147-nt-long *Bclaf1*^exon11^ encodes peptides across the third low-complexity region (LCR3) at the C-terminus, a shorter BCLAF1 isoform was annotated (Uniprot ID: F8WI22, Fig. 6G). The high content of threonine and asparagine residues in LCR3 as well as the glycine-arginine repeats within AA802-809, gave rise to multivalent interactions between BCLAF1 and its cofactors, possibly affecting the distribution pattern of BCLAF1.

UHM assist U2AF1/2 dimerization, and is responsible for U2AF1 substrate recognition [63] (Fig. S6B). UHM-depleted U2AF1 was unable to interact with U2AF2 or other exonic splicing factors, including SRSF2 (Fig. S6D-E). While the introduction of U2AF1 led to *Bclaf1*^exon11^ exclusion in FGOs, ectopic U2AF1^ΔUHM^ did not alter its inclusion preference compared to the control group (Fig. S6C). U2AF1 presented higher affinity towards the intronic region upstream *Bclaf1*^exon11^ to the one ahead of *Bclaf1*^exon12^, enclosing the corresponding PPTs as revealed by RNA-immunoprecipitation (RIP) analysis (Fig. 6I). The U2AF1^ΔUHM^ isoform, however, presented similar affinity for both intronic regions (Fig. 6J). Thus, U2AF1, which relies on the UHM, is responsible for substrate selection and *Bclaf1* mRNA processing. An adequate abundance and suitable forms of BCLAF1 variants are essential for their positioning and pre-implantation development.

### Massive alternative exons are manipulated through PPT-U2AF selection during zygotic splicing activation

Several ZGA gene products also undergo alternative exon processing upon U2AF1 overexpression. Expressed in the early follicle stages, XPO1 maintains low abundance in oocytes during meiotic maturation [64]. After fertilization, *Xpo1* accumulated in abundance and was processed into its full-length form (Fig. 4I and Fig. S7A). In ectopic U2AF1-expressing 2-cells, *Xpo1*^exon13^ tended to be excluded. Thus, a frameshift occurred and a truncated, only N-terminus-maintaining XPO1 isoform was translated upon *Xpo1*^exon13^ exclusion (Fig. S7B-C). The loss of HEAT repeats at the C-terminus can hamper nuclear export driven by XPO1 [65]. Similarly, *Gpbp1*^exon5^ was not selectively included in excessive U2AF1-expressing 2-cell embryos (Fig. S7D-E). The introduction of UHM-lacking U2AF1 did not alter the composition of *Gpbp1* variants, suggesting that U2AF1 plays a vital role in exon usage (Fig. S7F).

Besides *Bclaf1*, other genes were found to obey U2AF1-inducible, 2-cell preferred exon skipping, including *Tcp1* (Fig. S7G-I). Almost all *Tcp1* exon 2 was attached to exon 3 in FGOs, whereas excessive U2AF1 enhanced *Tcp1*^exon2^ exclusion in 2-cell embryos. TCP1 serves as a component of the TCP1-ring complex (TRiC), reported to function in sperm-zona pellucida interaction, and is thus important for fertilization [66, 67]. When exon 2 was lost, a frameshift occurred and *Tcp1* encountered a premature termination codon, resulting in a 26-AA-long peptide. Although PPTs are primarily recognized by U2AF2, the downstream AG-bound U2AF1 participates in exon determination [68]. A few exons presented U2AF1-inhibited, 2-cell preferred inclusion (Fig. 5J, quadrant Ⅱ). A lower level of *Ezh2*^exon14^ was detected in U2AF1-hyperexpressing 2-cell embryos than in the control cohort (Fig. S7J-K and S7M). A lower molecular weight EZH2 protein isoform was also detected (Fig. S7L). Since the PPT upstream of *Ezh2*^exon14^ was relatively longer but contained more pyrimidines (TCATTTCCCATCTCCCT, as predicted in the genome) than the one upstream of *Ezh2*^exon15^ (TACATTTATCCCT), U2AF1 displayed higher affinity towards the intronic region upstream of *Ezh2*^exon14^ than the one upstream of *Ezh2*^exon15^ (Fig. S7N). Depletion of UHM impaired U2AF1 interactions in both regions (Fig. S7O). Collectively, relatively low U2AF1 abundance is vital for massive pre-mRNA processing. Otherwise, in the case of U2AF1 hyperexpression, disorderly spliced transcripts could be subjected to degradation, translated into dysfunctional proteins, and thus be detrimental to physiological conditions.

## Discussion

The transcriptomic transition of oocytes and pre-implantation embryos is derived from a combination of events, including maternal-zygotic-transited transcription, maternal mRNA removal, and altered pre-mRNA splicing. Despite the poor processing of gene products of promiscuous transcription in zygotes, ZSA governs pre-mRNA splicing and generates numerous variants accompanying ZGA at the 2-cell stage [10, 50]. Defective ZSA machinery, including the direct inhibition of spliceosome components [10], and decreased amounts of splicing factors in aged oocyte-derived embryos [56], results in a disordered transcriptome and impaired embryonic development. Therefore, fine regulons are required to safeguard the ZSA process and serve as RNA-variant monitors.

**Fig. 7:**
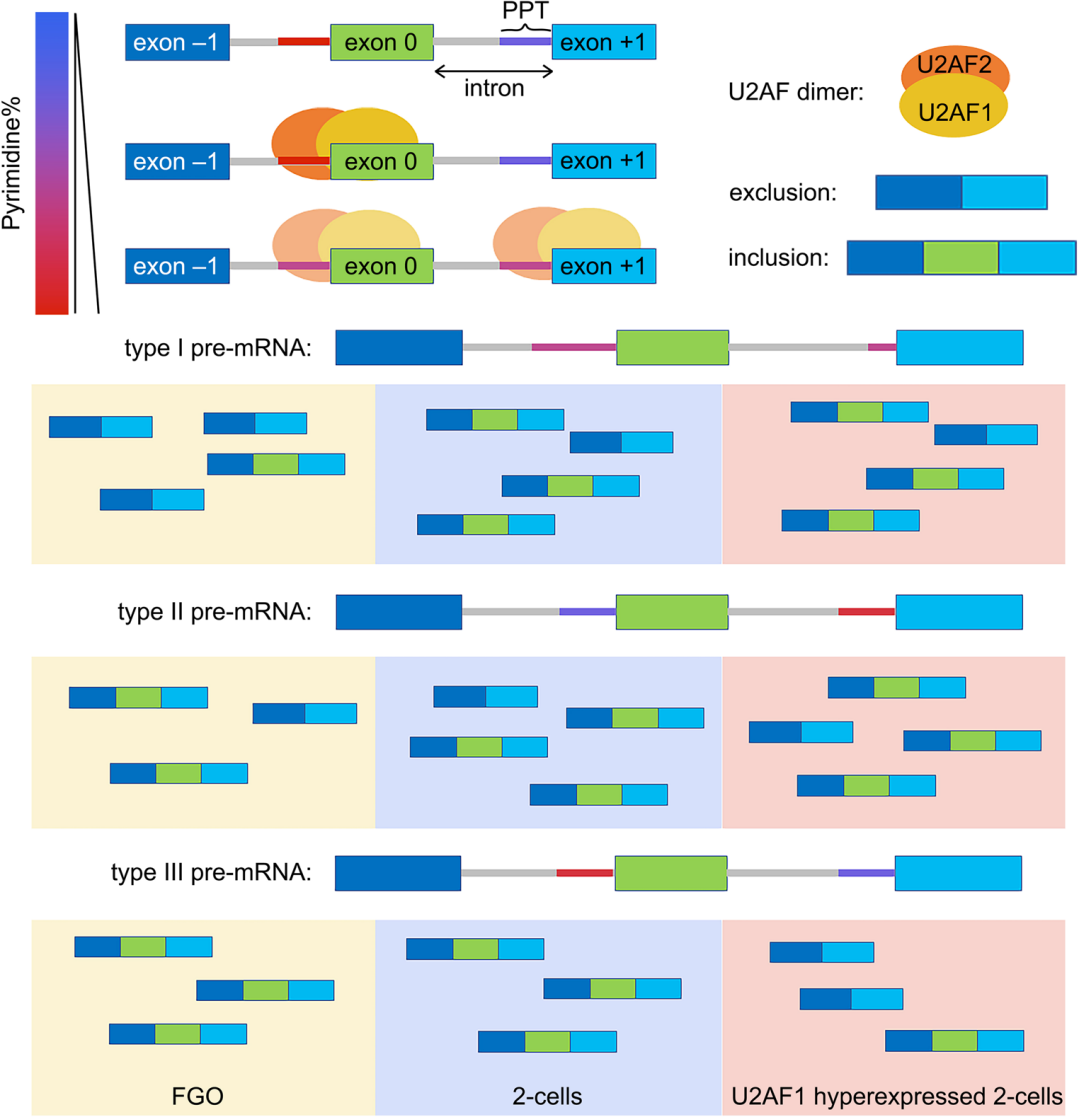
Scheme of U2AF1-dependent exon selection in mouse 2-cell embryos. Intronic elements complements the logic of exon usage. U2AF homologues form dimers and participate in splice site decision. The affinity of U2AF1 for splice sites is highly sensitive to the upstream polypyrimidine tracts (PPTs). In 2-cell embryos, U2AF1 is hypo-expressed, leading alternative exons following longer, less pyrimidine-containing PPTs to be included comparing to oocytes (types Ⅰ and Ⅱ, e.g. *Ezh2*^exon14^). U2AF1 overexpression enables U2AF to recognize strong PPTs with higher pyrimidine contents in 2-cells, promotes exclusion of their downstream exons (type Ⅲ, e.g. *Rbm5*^exon8^ and *Bclaf1*^exon11^). Excessive U2AF1 results in defective zygotic splicing activation (ZSA), disrupted the zygotic mRNA reserve, and thus impairs preimplantation development. The alternative exon (exon 0), and its upstream (exon − 1) and downstream ones (exon + 1) are represented by green, dark blue and light blue boxes, respectively. The pyrimidine abundance of the PPTs is indicated by a gradient from blue to red. Three types of pre-mRNA configurations are presented: Type Ⅰ, PPTs upstream exons 0 and + 1 earn similar pyrimidine content, but the PPT upstream exon 0 is longer. Type Ⅱ and Ⅲ, the PPTs upstream exons 0 and + 1 are similar in length, but PPTs upstream the type Ⅱ exon +1s and the type Ⅲ exon 0s contain more pyrimidines

A group of factors have been demonstrated to be essential for splicing in mouse oocytes, e.g. RTCB for intron removal, PABPN1 for 3′-UTR selection, and YTHDC1 for cleavage [24, 25, 51]. In this study, by comparison to FGOs, a majority of alternative exons were excluded in mRNA variants in 2-cell embryos (Fig. 1). In embryos at different stages, blastomeres undergoing ZGA also harbor the most alternative exons across species [12]. Such alternative exons (exon 0 s) were commonly downstream of less pyrimidine-incorporated PPTs than the corresponding downstream ones (exon +1s). Alternative exons included in 2-cells tended to follow longer PPTs than exon +1s and were not sensitive to pyrimidine concentrations. A previously reported 3′ splice site recognizer, U2AF1, maintained in low abundance in pre-implantation embryos, was responsible for ZSA processing. U2AF1 was translationally active during meiotic maturation and thereafter maintained a low protein level in early embryos (Fig. 2 and Fig. S4). Upon U2AF1 hyperexpression through in vitro transcribed mRNA microinjection, numerous gene transcripts were downregulated, and massive ASEs occurred, especially in SE-containing cassettes in 2-cell embryos (Figs. 4 and 5). More than 70% of alternative exons were selectively excluded upon ectopic U2AF1 expression. Although U2AF1 primarily recognizes AG dinucleotides at the 3′ splice site, the binding affinity of U2AF1 for substrates was sensitive to PPT pyrimidine concentration rather than lengths (Fig. 7). The conjugation of PPT-bound U2AF2, the dimerization of U2AF1/2, indirect association of U2AF1 and PPTs, and excessive U2AF1 integrated in 2-cell exon decision. 3′ splice sites could even be dispensable for immature mRNAs with strong PPTs, as we found several exons of *Nrf1*, *Gm6264*, *Cfap97* and *Ceacam20* that did not follow AG at their 5′-end [69]. Intronic regions following more pyrimidine-containing PPTs were preferentially bound by U2AF1, leading to the exclusion of their downstream exon 0 s, *e.g. Bclaf1*, in U2AF1 hyperexpression 2-cells (Fig. 6). Moreover, high levels of U2AF1 expression disturbed the deposition of zygotic variants, with 41.32% of skipping cassettes further enhanced and 54.55% of SE cassettes repressed in 2-cell embryos.

Although cleavage and ligation occur only at splice sites, *cis*-regulatory elements and *trans*-splicing factors also govern splicing decisions. Nuclear speckles, which enrich various mRNA processing factors and function as hubs for polyadenylated RNAs, are thought to participate in pre-mRNA splicing [70]. Splicing factors are often observed in nuclear speckles, especially SR proteins enriched in serine and arginine residues (SRSF1 [46], SRSF2 [71], and SRRM2 [48] for instance). Accordingly, SR proteins mainly serve as exon interactors because their binding motifs are enriched in exons [72]. However, hnRNPs, known as intronic splicing regulators, are not enriched in, or are even sequestered out of, nuclear speckles [54, 73–75]. An interface splicing model suggests that pre-mRNAs should be finely positioned at the nuclear speckle/nucleoplasm interface, with SR-bound exonic regions within the nuclear speckles, and introns bound by hnRNPs outside the speckles [53]. Oocytes are an ideal model for research concerning transcription and pre-mRNA processing because growing oocytes are transcriptionally active, whereas such transcription processes cease in SN-stage oocytes [76]. In this study, we identified U2AF1 as another splicing regulator residing outside nuclear speckles in mouse oocytes (Fig. 3). U2AF1 was mainly dispersed in the nucleoplasm in oocytes. A small number of low-density U2AF1 nuclear regions were observed by immunofluorescence microscopy in both GOs and FGOs, whereas nuclear speckles were observed in such regions. Its homologue, U2AF2, displays a divergent pattern. U2AF1/2 were previously reported to form dimers and collaborate in 3′ splice site determination [45, 77]. Alternative exons controlled by U2AF1/2 in HEK293 cells are largely shared, with only a few very short exons, e.g. an 18 nt-long exon of *MAPK8IP3* differed [45]. In transcriptionally active oocytes, U2AF2 was not enriched or depleted from the speckles and was simply dispersed within the nucleoplasm (Fig. S3). In SN-stage FGOs, U2AF2 was observed in the speckles. A recent study reported that U2AF1 engages in co-transcriptional splicing by traveling between the transcription machinery and the U2AF1/2 heterodimer [78]. Although speckles in oocytes and early embryos share multiple components, the also exhibit distinct feature. Speckle formation in mouse 2-cell embryos was time-sensitive, temporally coupling the ZGA transcription program. During this period, U2AF1 remained at low abundance, diffusely distributed in the nucleoplasm without forming cavities (Fig. S4). Exogenous U2AF1 supplementation could not change this pattern in 2-cells. It is possible that, under robust transcription, U2AF1/2 are recruited by nascent mRNAs around the transcription locus in 2-cell embryos and pre-mRNAs are processed co-transcriptionally. When transcription ceases, particularly in SN-staged FGOs, immature transcripts are packaged into speckles together with PPT-bound U2AF2, whereas U2AF1 remains in the outer layer of the speckles and governs post-transcriptional processing. Such spatial compartmentation enables splice site positioning and could probably strengthen splicing decision. Taken together, we proposed that U2AFs served as splicing regulators, functioning in both co- and post-transcriptional program, and further ensuring fine compartmentation for post-transcriptional splicing.

Alternative splicing, which involves the use of alternative exons, results in the production of a variety of mRNA isoforms. Inclusion or exclusion of certain exons affects mRNA metabolism in various aspects, i.e*.* changing protein isoforms, introducing frameshift mutations, and influencing stability. The types of mRNA variants, including the proportions of subspecies, differ between oocytes and embryos [79]. For instance, full-length *Ezh2* mRNA was predominantly found in 2-cell embryos, whereas exon 14-lacking variants accounted for only a minority. Equal amounts of exon 11-inclusive and exclusive *Bclaf1* variants were detected in 2-cell embryos, and FGOs harbored more full-length *Bclaf1* transcripts. Altered products of only one gene may be detrimental to embryonic development [10, 15, 20]. Nearly half of ZGA-accompanied exon alternations result in frame shifts, of which 80% disrupt the open reading frames in 2-cell mouse embryos and in 8-cell human embryos [12]. These transcripts trigger nonsense-mediated decay and are cleared. Ectopic U2AF1 expression enhanced the interaction between U2AF1 and high-pyrimidine PPTs and induced exon exclusion in 2-cell embryos, thereby generating multiple mRNA variants as well as their cognate encoded proteins (Fig. 6 and S7). In concert with U2AF2, U2AF1-bound 3′ splice sites could be promoted, even at weak sites [80, 81]. This phenomenon was even more notable for transcripts carrying stronger PPTs with more incorporated pyrimidines. Transcripts encoded by ZGA genes (*i.e. Xpo1* and *Gpbp1*) were also affected by U2AF1. Although we demonstrated that BCLAF1 isoforms play different roles during embryogenesis, our mRNA microinjection assay amplified the effects of a single gene. Hence, excessive U2AF1 impairs early embryonic development through splicing dysregulation of massive pre-mRNAs, besides *Bclaf1* itself.

A pair of MXEs, exons 3a/b were evolutionarily conserved in U2AF1 and encoded both functional proteins [43]. Although the corresponding peptides are almost identical, U2AF1^3A/3B^ differ in 7 amino acids. Since U2AF1^3A^ served as the predominant form in most cell types and across species, we cloned U2AF1^3A^ and analyzed its role in early embryogenesis. Subsequent research on U2AF1^3B^ would provide more details in U2AF1 function. Also, the contribution of U2AF2 to exon selection in 2-cell embryos under excessive U2AF1 ought to be determined. How does the composition of PPTs affect U2AF recognition, and whether if the U2AF1/2 ratio contributes to splice site determination are to be examined.

In summary, our study revealed the role of U2AF1 in early embryonic exon selection. PPTs containing more pyrimidines were preferentially recognized by U2AFs, leading to the exclusion of their downstream exons. In 2-cell embryos, U2AF1 was maintained at low abundance, assisting alternative exons following longer, but low-pyrimidine-ratio PPTs. Meanwhile, exclusion of exons downstream of the high pyrimidine ratio PPTs was restrained. Through dimerization with U2AF2 and indirect association with PPTs, ectopic U2AF1 expression disrupted exon usage in 2-cell embryos, dysregulated the ZSA process, disturbed the transcriptome status, and impaired mouse pre-implantation embryogenesis.

## Materials and methods

### Animals

Wild-type (WT) ICR mice were obtained from the Zhejiang Academy of Medical Science, China. Mice were bred under specific pathogen-free (SPF) conditions in a controlled environment at 20–22 °C, 50–70% humidity, and within a 12/12 h light/dark cycle. Food and water were provided *ad libitum*. All animal experiments were conducted in accordance with the guidelines and regulations of Zhejiang University, with an approved experimental protocol (ZJU20250080) by the Institutional Animal Care and Research Committee of Zhejiang University.

### Oocyte collection and in vitro culture

Fully grown oocytes (FGOs) and growing oocytes (GOs) were harvested from female mice at postnatal day 21–23 (PD21-23), pre-treated with pregnant mare serum gonadotropin (PMSG, Ningbo Sansheng Pharmaceutical Co., Ltd., P. R. China), and PD14-16, respectively. Oocytes were collected in M2 medium (Nanjing Luanchuang Life Technology, P. R. China, M01-B) plus 1% penicillin-streptomycin solution (ZETA Life, BM0001), and cultured in M16 medium (Sigma-Aldrich, M7292) minidrops, covered with mineral oil (DEWIN, 9902). In vitro cultured oocytes were kept in a humidified incubator, at 37 ℃ in 5% (v/v) CO_2_ atmosphere.

### Superovulation, fertilization and embryo in vitro culture

Female mice at PD26-28 were intraperitoneally injected with 5 IU PMSG. These mice were further treated with 5 IU human chorionic gonadotropin (hCG, Ningbo Sansheng Pharmaceutical Co., Ltd., P. R. China) after 48 h post PMSG application, and mated with 12-week-old males. Successful mating was confirmed by observed vaginal plugs. Embryos were harvested from the oviducts 22 h post hCG injection, and in vitro cultured in potassium simplex optimized medium (KSOM, Nanjing Luanchuang Life Technology, P. R. China, M03-AA) minidrops covered with mineral oil, in a humidified incubator, at 37 ℃ in 5% (v/v) CO_2_ atmosphere.

### Cell culture

Human ovarian cancer cell, SK-OV-3, was cultured in Dulbecco’s Modified Eagle Medium (DMEM, GIBCO) plus 10% fetal bovine serum (FBS, ZETA Life) and 1% penicillin-streptomycin solution in a humidified incubator, at 37 °C in 5% (v/v) CO_2_ atmosphere.

### Plasmid construction and in vitro transcription

The corresponding cDNAs, including *U2AF1*, *Bclaf1* and their variants, were subcloned into a mCherry-tagged expression vector (pDEST). The expression vectors were linearized with HindⅢ, and subjected to in vitro transcription using a T7 mMESSAGE mMACHINE kit (Invitrogen, AM1344). The transcribed RNA products were polyadenylated using a poly(A) tailing kit (Invitrogen, AM1350), and extracted and purified through lithium chloride precipitation.

### Microinjection of oocytes and zygotes

The microinjection experiments were performed using an Eppendorf TransferMan NK2 micromanipulator. Zygotes at 23–25 h post hCG injection were kept in M2 medium. To validate the positioning of BCLAF1 and its variant, FGOs were cultured in M2 medium plus 2 µM milrinone (Sigma, 475840) for spontaneous germinal vesicle breakdown inhibition. In vitro transcribed mRNAs were diluted to 800 g mL^− 1^ as described in Plasmid construction and in vitro transcription section. Approximately 5–10 pL of the indicated mRNA was microinjected into the zygote and the oocyte cytoplasm. RNAs transcribed from the pDEST-mCherry empty vectors were applied as the negative control. Oocytes and embryos were cultured in M16 and KSOM medium covered with mineral oil, respectively, at 37 °C in 5% (v/v) CO_2_ atmosphere.

### RNA extraction, reverse transcription, and quantitative real-time polymerase chain reaction (qRT-PCR)

RNAs were extracted from the indicated mouse tissues using the Invitrogen Ambio TRIzol reagent (Thermo Fisher Scientific) according to the manufacturer’s instructions. Turbo™ Dnase (Thermo Fisher Scientific) was applied to remove DNAs. The RNAs were reverse-transcribed with random primers using M-MLV Reverse Transcriptase (Invitrogen, 28025). Oocytes and embryos were collected and lysed directly with 0.2% Triton X-100 supplied with 4 IU recombinant RNase inhibitor (RRI, Takara, 2313). The oocytic and embryonic RNAs were reverse-transcribed with random primers employing PrimeScript™ II Reverse Transcriptase (Takara, 2690), according to the manufacturer’s instructions.

The cDNAs were subjected to qPCR using Universal SYBR Green Fast qPCR Mix (ABclonal, RK21203) and a Bio-Rad CFX96 Touch Real-Time PCR system. Relative mRNA abundance was calculated and compared with the respective cycle threshold (Ct) values. The corresponding primers of the indicated transcripts were listed in Table S6.

### Library construction and RNA sequencing

The RNAs isolated from U2AF1-hyperexpressing 2-cell embryos and the control group were subjected to RNA-seq analyses using the Smart-seq2 method. External RNA Controls Consortium (ERCC spike-in control mix, Invitrogen, 4456740) molecules were added to the samples. Sequencing libraries were then constructed with 0.5 ng cDNA using the TruePrep DNA Library Prep Kit V2 for Illumina (Vazyme, TD503) according to the manufacturer’s instructions. Barcoded libraries were pooled and sequenced on the Illumina NovaSeq 6000 platform in the 151 bp paired-end mode.

Raw reads were trimmed to remove low-quality bases and adaptor sequences using Trim Galore v0.6.7. Reads were further mapped to the mouse genome (mm10) using STAR v2.7.10a [82]. Uniquely mapped reads were applied for gene expression quantification using FeatureCounts v2.0.2 [83]. Gene expression abundance was analyzed using the DESeq2 R package, and fragments per kilobase of transcript per million mapped reads (FPKM) was calculated to validate gene expression and normalized to gene length and sequencing depth. For differentially expressed genes (DEGs), an adjusted *P*-value of < 0.05, and fold change (FC) of U2AF1-hyperexpression/mCherry-microinjection > 1.5 in 2-cell embryos was considered statistically significant, and vice versa.

### Alternative splicing event analysis

The alternative splicing events (ASEs) between WT FGOs and 2-cell embryos, as well as U2AF1-hyperexpressed and controlled 2-cell embryos, were analyzed by replicate multivariate analysis of transcript splicing (rMATS) [60]. Five subcategories of ASEs, including alternative-5′ spliced sites (A5SSs), alternative-3′ spliced sites (A3SSs), skipped exons (SEs), mutually exclusive exons (MXEs) and retained introns (RIs) were considered. Only events with false discovery rate (FDR) < 0.05 and IncLevelDifference over 0.1 or below − 0.1 were designated significant events. The ASEs were listed in Tables S1 and S4.

### Immunofluorescence and U2AF1 cavity measurement

Oocytes and embryos were fixed in 4% paraformaldehyde (PFA, Sigma-Aldrich, 158127) in phosphate buffered-saline (PBS) at 25 ℃ for 30 min. The samples were permeabilized in PBS containing 0.5% Triton X-100 at 25 ℃ for 30 min, and blocked with 1% bovine serum albumin (BSA, Sangon Biotech, A3311) at 25 ℃ for 30 min. Samples were incubated with the indicated primary antibodies according to Table S7, diluted in the blocking solution, at 4 ℃ overnight, and then incubated with Alexa Fluor 488-, 594-, and 647-conjugated secondary antibodies, and 4’, 6-diamidino-2-phenylindole (DAPI, Molecular Probes) at 25 ℃ for 30 min. The slides were imaged using a Zeiss LSM880 confocal microscope (Germany). For U2AF1 cavity measurement, Z-stack acquisition was performed to ensure complete cavity profile captured. Properties of the cavities were conducted on the largest cross-sections. A threshold was set from 0.25 to 25 µm^2^, avoiding false-positive signals. Quantification of the indicated IF signals was carried by Image J.

### Histological analysis

Ovaries collected from PD28 female mice were subjected to histological analyses. For immunohistochemistry analysis, ovaries were fixed in 4% formalin in PBS at 4 ℃ overnight, dehydrated, embedded in paraffin and sectioned at 5 μm. The ovarian sections were then deparaffinized and rehydrated, probed with the primary antibodies at 4℃ overnight as listed in Table S7, rinsed and incubated in biotin-labeled secondary antibodies at 25 ℃ for 30 min, following the instruction of the VECTASTAIN ABC kit and 3,3’-diaminobenzidine peroxidase substrate kit (Vector Laboratories).

For immunofluorescent analysis, ovaries were embedded optimal cutting temperature compound (OCT, Wuhan Seville Biotechnology Co., Ltd., P. R. China, G6059) and sectioned at 5 μm. The sections were fixed in 4% PFA, permeabilized, blocked with 10% goat serum (ZSGB-Bio, ZLI-9065) and incubated with the indicated primary antibodies. Images of the slides were acquired using an epifluorescence microscope (Nikon Eclipse 80i, Japan).

### Immunoprecipitation

SK-OV-3 cells were collected, and lysed 48 h post transfection of the indicated plasmids. Cell lysates were collected after centrifugation and subjected to immunoprecipitation (IP) analyses using anti-FLAG affinity gels (A4596; Sigma-Aldrich, St. Louis, MI, USA). Bead-bound proteins were collected after 4 °C incubation for 2 h and eluted with SDS sample buffer for western blot analysis.

### Ribonucleoprotein immunoprecipitation (RIP) assay

Endogenous U2AF1-bound RNAs were isolated in accordance to previously reported protocols [84], enriched by Protein A Sepharose 4 Fast Flow Bead (GE Healthcare)-conjugated U2AF1 antibody, as listed in Table S7. Rabbit (DA1E) mAb IgG XP Isotype (Cell Signaling Technology, 3423) was applied as the control. For UHM-lacking U2AF1-interacting RNAs, lysates of FGOs microinjected with Flag-tagged U2AF1^ΔUHM^ were collected and incubated with anti-FLAG affinity gels. After incubation at 4 °C for 4 h, beads were collected and rinsed, and the bead-bound RNAs were extracted using RNeasy Mini Kit (QIANGEN, 74004). These RNAs were then reverse transcribed and analyzed as described before in the RNA extraction, reverse transcription, and quantitative real-time polymerase chain reaction (qRT-PCR) section.

### Western blotting analysis

Oocytes and embryos were collected and lysed within a β-mercaptoethanol containing SDS loading buffer. After heating at 95 °C for 10 min, the lysates and the eluted bead-bound proteins were separated through SDS-PAGE gel electrophoresis, and transferred to PVDF membranes (Millipore Crop., IPVH00010). The membranes were blocked in TBST with 5% noon-fat milk (m/v, Sangon Biotech, A600669) at 25 °C for 30 min, and probed with the indicated primary antibodies at 4 °C overnight. HRP-conjugated secondary antibodies were further applied at 25 °C for 40 min. The antibodies and the dilution index used were listed in Table S7.

### Statistical analysis

Statistical data are presented as Mean ± standard error of the mean (SEM) or Mean ± standard deviation (SD) as indicated. Each experiment was repeat at least thrice. For qRT-PCR results, gene expression abundance was normalized to the *Actin* expression levels, and the RIP-enriched RNAs were normalized to the IgG and the control groups, as indicated. Two-tailed unpaired Student’s *t*-tests were performed to compare the results of the two indicated experimental groups. Results of which *P <* 0.05 were considered statistically significant. “*n.s.*” indicated not significant.

## Supplementary Information

Below is the link to the electronic supplementary material.

Supplementary Material 1 (DOCX 28.0 KB)

Supplementary Material 2 (XLSX 3.10 MB)

Supplementary Material 3 (XLSX 32.0 KB)

Supplementary Material 4 (XLSX 3.38 MB)

Supplementary Material 5 (XLSX 2.96 MB)

Supplementary Material 6 (XLSX 42.5 MB)

Supplementary Material 7 (DOCX 2.70 MB)

## Data Availability

The raw RNA-seq datasets generated in current study were deposited on the Genome Sequence Archive in National Genomics Data Center [85, 86]. The data was accessible at https://ngdc.cncb.ac.cn/gsa with the accession number CRA033200. Previously published RNA-seq data [58, 59, 87–89] used in this work were from NCBI GEO accession number GSE254288 (mouse FGOs), and GSE165782 (mouse embryos), and Genome Sequence Archive accession number CRA001613 (mouse oocytes during folliculogenesis).
