# Supplementary material for: U2AF1 modulates alternative exon selection and guards zygotic splicing activation in mouse preimplantation embryogenesis
Source: Cell Mol Life Sci. 2026 Apr 16;83(1):226. doi: 10.1007/s00018-026-06197-y (PMC13199581; doi:10.1007/s00018-026-06197-y)
Supplement: Supplementary file 1 — Supplementary Material 1 (DOCX 28.0 KB) [file 18_2026_6197_MOESM1_ESM.docx]

**Supplementary tables**

**Table S1. Alternative splicing events comparing wild-typed fully-grown oocytes (FGOs) and 2-cell embryos. (In a separated xlsx file).**

**Table S2. Predicted polypyrimidine tracts (PPTs) upstream exon 0s and +1s of the TOP50 skipped exon-containing cassettes comparing wilde-typed FGOs and 2-cell embryos with highest and lowest IncLevelDifference. (In a separated xlsx file).**

**Table S3. FPKMs of transcripts detected in ectopic mCherry-U2AF1-expressing 2-cell embryos comparing to the control group. (In a separated xlsx file).**

**Table S4. Alternative splicing events upon U2AF1 hyperexpression in 2-cell embryos. (In a separated xlsx file).**

**Table S5. Predicted PPTs upstream exon 0s and +1s of SE-containing cassettes in 2-cell embryos upon U2AF1 hyperexpression. (In a separated xlsx file).**

**Table S6. Primer sequences**

| **Primer name** | **Gene targeted** | **Application** | **Sequence (5'-3')** |
| --- | --- | --- | --- |
| Actin-F | *Actin* | Real-time PCR | GCTCTTTTCCAGCCTTCCTT |
| Actin-R |  | Real-time PCR | GTACTTGCGCTCAGGAGGAG |
| Oosp3 exon1-F | *Oosp3* | gel electrophersis | TGCTTCAGGATTGCTGCTGCT |
| Oosp3 exon3-R |  | gel electrophersis | TGGGAATGACACACTGAAGCTGAA |
| Ezh2-F | *Ezh2* | Real-time PCR | AGTGACTTGGATTTTCCAGCAC |
| Ezh2-R |  | Real-time PCR | AATTCTGTTGTAAGGGCGACC |
| Ezh2 exon14-F |  | Real-time PCR | ACAACTATCAACCCTGTGACCATCCAC |
| Ezh2 exon14-R |  | Real-time PCR | TGTGCTATCACACAAGGGCACGA |
| Ezh2 intron13-F |  | Real-time PCR | CCAGTCAAGGATGTTCCAGAGGTAGTG |
| Ezh2 exon14-R1 |  | Real-time PCR | TGTGCTATCACACAAGGGCACGA |
| Ezh2 intron14-F |  | Real-time PCR | AGGATGTGGATGATGAGGGTGATTCTT |
| Ezh2 exon15-R1 |  | Real-time PCR | ACCGACATCCAGGAAAGCGGTT |
| Ezh2 exon13-F |  | gel electrophersis | TGGGCTGCACACTGCAGAAAGA |
| Ezh2 exon15-R |  | gel electrophersis | AGGTCAGGGTCACACTCTCGGACA |
| Wee2 exon1-F | *Wee2* | gel electrophersis | TCACTGGCTTGTAACCAAGAACCT |
| Wee2 exon2-R |  | gel electrophersis | TCTCTGGTAGGAGGCACGGGA |
| Ptma exon2-F | *Ptma* | gel electrophersis | AGAGATGCACCTGCCAATGGGA |
| Ptma exon4-R |  | gel electrophersis | TCATCATCCTCAGCTACCCGCT |
| U2af1-F | *U2af1* | Real-time PCR | GAGAAGTACGGGGAAGTCGAG |
| U2af1-R |  | Real-time PCR | ACGTCGAAACTTGACATACACG |
| U2af1 exon3a-F |  | Real-time PCR | CCATTGCCCTCTTGAACATTTACCG |
| U2af1 exon3a-R |  | Real-time PCR | AGCGCAAACCGTCAGCAGACTG |
| U2af1 exon3b-F |  | Real-time PCR | CCATCTTGATTCAAAACATCTATCGTAATCC |
| U2af1 exon3b-R |  | Real-time PCR | GTGTGAGCCGTCAGCCGTCTG |
| U2af2-F | *U2af2* | Real-time PCR | ACAGGAAGCGTAGTCACAGTC |
| U2af2-R |  | Real-time PCR | TTCGTCGTCTCCTATCCCGAG |
| Zscan4d-F | *Zscan4d* | Real-time PCR | GCAGATGCCAGTAGACACCA |
| Zscan4d-R |  | Real-time PCR | GGCATCAAGAGGGAATTGAA |
| Zscan4c-F | *Zscan4c* | Real-time PCR | GCCTTATGTCTGTTCCCTATGT |
| Zscan4c-R |  | Real-time PCR | CAGTCTCTGCTGAGGATGTTAG |
| Ubtfl1-F | *Ubtfl1* | Real-time PCR | ATCCTGACCGACCCAAAAGAC |
| Ubtfl1-2 |  | Real-time PCR | GCTGTGCGTTGCTGTACTTAG |
| Zfp352-F | *Zfp352* | Real-time PCR | TGACCCAAAATACAGCCATGTG |
| Zfp352-R |  | Real-time PCR | TGACTGTTGTAGGAGGTCCAG |
| Xpo1-F | *Xpo1* | Real-time PCR | TGGAGAAGTAATGCCGTTCATTG |
| Xpo1-R |  | Real-time PCR | CCCACACTTGATTAGGGAGTAGC |
| Xpo1 exon11-F |  | gel electrophersis | TGGCAGCTGAGCTCTACAGAGAGA |
| Xpo1 exon13-R |  | gel electrophersis | CTTCCATGACCATTCTGTACCATTCA |
| Dppa4-F | *Dppa4* | Real-time PCR | AGTCAACCTAGCACGGCTC |
| Dppa4-R |  | Real-time PCR | TCCTGGCGTCTCAGTGTCT |
| Tead4-F | *Tead4* | Real-time PCR | GGAGTATGCCCGCTATGAGA |
| Tead4-R |  | Real-time PCR | TCCTGTGTGTCTCGGTTGGT |
| Ccnb1ip1-F | *Ccnb1ip1* | Real-time PCR | GGTACACCAGGAGCGTCTCTA |
| Ccnb1ip1-R |  | Real-time PCR | GCTTTTGGTACTGGCGATTACG |
| Sf3b1-F | *Sf3b1* | Real-time PCR | GTGGGCCTTGATTCCACAGG |
| Sf3b1-R |  | Real-time PCR | GGCTTCTTCTGACCGAGCAA |
| Srsf1-F | *Srsf1* | Real-time PCR | TCTCACGAGGGAGAAACTGC |
| Srsf1-R |  | Real-time PCR | CTGCTACGGCTTCTGCTACG |
| Srsf2-F | *Srsf2* | Real-time PCR | CGCGCTCCAGATCAACCTC |
| Srsf2-R |  | Real-time PCR | CTTGGACTCTCGCTTCGACAC |
| Srsf3-F | *Srsf3* | Real-time PCR | GCGCAGATCCCCAAGAAGG |
| Srsf3-R |  | Real-time PCR | ATCGGCTACGAGACCTAGAGA |
| Srsf5-F | *Srsf5* | Real-time PCR | CTTCAAGGGTTACGGACGGAT |
| Srsf5-R |  | Real-time PCR | GGGCATGTTCAATCGTCACC |
| Srrm2-F | *Srrm2* | Real-time PCR | TTTTGATCCTCAGAGACGTGC |
| Srrm2-R |  | Real-time PCR | GTTTCCGTTTCTTGGACTCAGA |
| Son-F | *Son* | Real-time PCR | TTTCAGGTCTTTCGTGGTCAGT |
| Son-R |  | Real-time PCR | GGTGGATTTGTTTCACCATTCAG |
| Pabpn1-F | *Pabpn1* | Real-time PCR | GGTGATTCCCAAACGAACCAACAG |
| Pabpn1-R |  | Real-time PCR | GTCGCTCTAGCCCGGCCC |
| HnrnpA1-F | *Hnrnpa1* | Real-time PCR | TGGAAGCAATTTTGGAGGTGG |
| HnrnpA1-R |  | Real-time PCR | GGTTCCGTGGTTTAGCAAAGT |
| Zc3h13-F | *Zc3h13* | Real-time PCR | ATCCCGAAGACCTAGCGTATT |
| Zc3h13-R |  | Real-time PCR | TGAAGGGCCATGTATGAACCT |
| Rbm5-F | *Rbm5* | Real-time PCR | CGGCGGGACTCAGATTACAAA |
| Rbm5-R |  | Real-time PCR | ACGGAGCATGATGGTCTTGC |
| Rbm5 exon7-F |  | gel electrophersis | GGTGATTCAAGGAAAGCACATTGC |
| Rbm5 exon9-R |  | gel electrophersis | GGACTGAGCAGACTCTGTGGTTC |
| Dnaja1-F | *Dnaja1* | Real-time PCR, gel electrophersis | ACCACTTACTACGATGTTTTGGG |
| Dnaja1-R |  | Real-time PCR | GCCCTCTTTAATCGCCTGCT |
| Dnaja1 exon3-R |  | gel electrophersis | CTTCACATTTGTCACAAATCACATTC |
| Dnaja1 intron 2-F |  | Real-time PCR | TTCGTGCCTTAAAGTCTACCGTAGCTGT |
| Dnaja1 exon3-R1 |  | Real-time PCR | TCTTTAATCGCCTGCTCCCCTCC |
| Dnaja1 intron 3-F |  | Real-time PCR | AAGATGTATAGTTTGAGGCTGGTCTTCCAT |
| Dnaja1 exon4-R1 |  | Real-time PCR | CTAAGGTCACTGAGAGCTGATGAACAACA |
| Cers5-F | *Cers5* | Real-time PCR | GCCTATCAAAGCAACTGGACTGGAGT |
| Cers5-R |  | Real-time PCR | CGACCAGAGAAATCTGATTCCGTAGC |
| Cers5 exon4-F |  | gel electrophersis | TCTGCTACGGAATCAGATTTCTCTGGTC |
| Cers5 exon7-R |  | gel electrophersis | GGGTCTGCGAAGTCATGCAGACA |
| Bclaf1-F | *Bclaf1* | Real-time PCR | ACTCACCACGAGATGAAAGACT |
| Bclaf1-R |  | Real-time PCR | TGTCCCAGCAAAAACTCCTCT |
| Bclaf1 exon11-F |  | Real-time PCR | TGCTGGGACAAATACTGGTCCAAAC |
| Bclaf1 exon11-R |  | Real-time PCR | CTTCTTGCTCTTTGGGGTATATTCTGGA |
| Bclaf1 intron 10-F |  | Real-time PCR | ACACACACACACACCACATACACTTGCT |
| Bclaf1 exon 11-R1 |  | Real-time PCR | TCCCAGCAAAAACTCCTCTGGCT |
| Bclaf1 intron 11-F |  | Real-time PCR | TGCATGACAGCTGTATGTTAGAGAATTGGT |
| Bclaf1 exon 12-R1 |  | Real-time PCR | GCCACGTTGAAAAGTACCACGACC |
| Gpbp1 exon4-F | *Gpbp1* | gel electrophersis | ATCGTTATGATGTGAGTCGTCGA |
| Gpbp1 exon6-R |  | gel electrophersis | TGATTTGGTTCTCTCTCATATTCAGGA |
| Tcp1 exon1-F | *Tcp1* | gel electrophersis | TTGTCTGCGGCCGTAGTGA |
| Tcp1 exon3-R |  | gel electrophersis | TCTTTGTCTTGCAGGTCAGCCA |

**Table S7. Antibody information**

| **Protein/Target name** | **Manufacture (catalogue number)** | **Application (working dilution)** | **Website link** |
| --- | --- | --- | --- |
| DDB1 | Abcam (ab9194) | western blotting (1:2000) | <https://www.abcam.cn/products/primary-antibodies/ddb1-antibody-ab9194.html> |
| EZH2 | Cell Signaling Technology (5246) | IF (1:200), wester blotting (1:1000) | <https://www.cellsignal.cn/products/primary-antibodies/ezh2-d2c9-xp-rabbit-mab/5246> |
| H3K27me3 | Cell Signaling Technology (9733) | IF (1:400) | <https://www.cellsignal.cn/products/primary-antibodies/tri-methyl-histone-h3-lys27-c36b11-rabbit-mab/9733> |
| U2AF1 | ABclonal (A1046) | IHC (1:200), IF (1:100), western blotting (1:1000) | <https://abclonal.com.cn/catalog/A1046> |
| U2AF2 | ABclonal (A4552) | IHC (1:200), IF (2:100), western blotting (1:1000) | <https://abclonal.com.cn/catalog/A4552> |
| SRSF2 | Sigma-Aldrich (SAB4200725) | IF (1:100) | <https://www.sigmaaldrich.cn/CN/zh/product/sigma/sab4200725> |
| HNRNPA1 | ProteinTech (11176) | IF (1:200) | <https://www.ptgcn.com/products/HNRNPA1-Antibody-11176-1-AP.htm> |
| Nanog | Cell Signaling Technology (8822) | IF (1:100) | <https://www.cellsignal.com/products/primary-antibodies/nanog-d2a3-rabbit-monoclonal-antibody/8822> |
| CDX2 | BioGenex (AM392-5M) | IF (direct use) | <https://biogenex.com/product/anti-cdx-2/> |
| FLAG | Sigma (F3165) | western blotting (1:3000) | http://www.sigmaaldrich.com/catalog/product/sigma/f3165?lang=zh&region=CN |
| HA | Cell Signaling Technology (3724) | western blotting (1:1000) | <https://www.cellsignal.com/products/primary-antibodies/ha-tag-c29f4-rabbit-mab/3724> |
| IgG | Cell Signaling Technology (3900) | endogenous RIP | [https://www.cst-c.com.cn/products/primaryantibodies/ rabbit-da1e-mab-igg-xp-isotypecontrol/ 3900?site-search-type=Products](https://www.cst-c.com.cn/products/primaryantibodies/rabbit-da1e-mab-igg-xp-isotypecontrol/3900?site-search-type=Products) |
