# Supplementary material for: U2AF1 modulates alternative exon selection and guards zygotic splicing activation in mouse preimplantation embryogenesis
Source: Cell Mol Life Sci. 2026 Apr 16;83(1):226. doi: 10.1007/s00018-026-06197-y (PMC13199581; doi:10.1007/s00018-026-06197-y)
Supplement: Supplementary file 7 — Supplementary Material 7 (DOCX 2.70 MB) [file 18_2026_6197_MOESM7_ESM.docx]

**Figure legends**

**
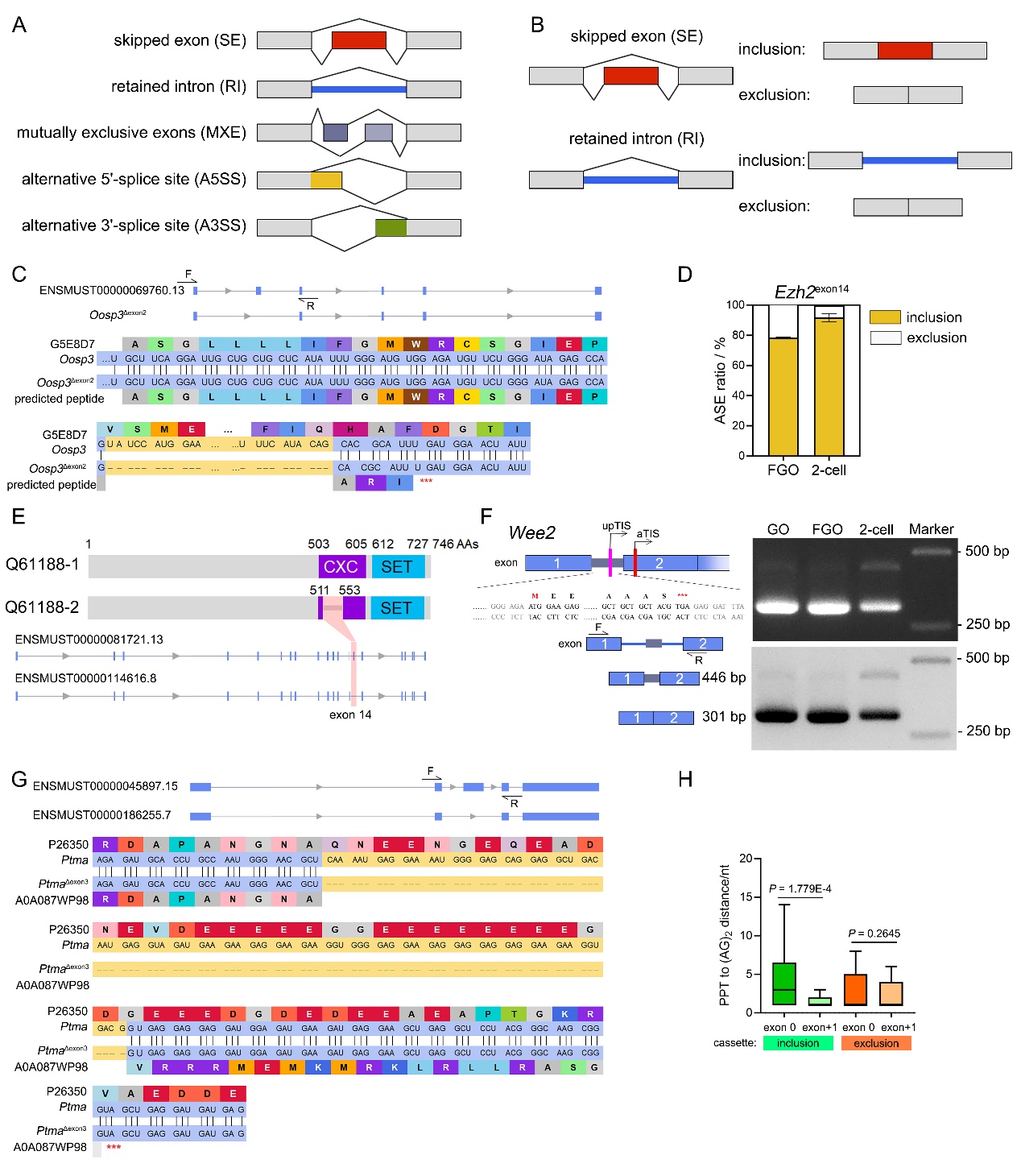
**

**Supplementary Figure.1 Mouse oocytes and 2-cell embryos harbor divert mRNA variants. A-B:** Graphic illustration of alternative splicing events (ASEs) and splicing outcomes. Five categories of ASEs, including skipped exon (SE), retained intron (RI), mutually exclusive exons (MXE), alternative 5'-splice site (A5SS) and 3'-splice site (A3SS) were analyzed (**A**). Alternatively spliced SE and RI are depicted in **B**. mRNA products either from inclusion or exclusion events are presented. **C:** Schematic illustration of full-length *Oosp3* and exon 2-lacking variants, and the predicted protein isoforms. *** indicates a premature termination codon. The localization of the primers is annotated. F, forward. R, reverse. **D:** Bar chart presenting *Ezh2*^exon14^ inclusion levels in transcriptome analysis. Mean ± SD. n = 3. FGO, fully grown oocyte. **E:** Comparison of EZH2 protein and RNA isoforms either containing exon 14 or not. The exon 14-encoding peptide is indicated in red. SET, Su(var)3-9, E(z), Trithorax domain. AA, amino acid. The Uniprot ID of the corresponding isoforms are indicated. **F:** Schematic illustration of *Wee2* transcript, its coding peptide sequences and PCR analysis of *Wee2* variants in growing oocytes (GOs), FGOs, and 2-cell embryos. bp, base pair. upTIS, upstream translation initiation site. aTIS, annotated translation initiation site. **G:** Schematic illustration of full-length *Ptma* and exon 3-lacking variants. **H:** The nucleotides between the predicted polypyrimidine tracts (PPTs) and the 3'-splice sites within the TOP 50 inclusive and exclusive cassettes, comparing the SE events in 2-cell embryos to the FGO group. The alternatively skipped exons are annotated as exon 0s, and the downstream ones are annotated as exon +1s. nt, nucleotide. Mean ± SEM.


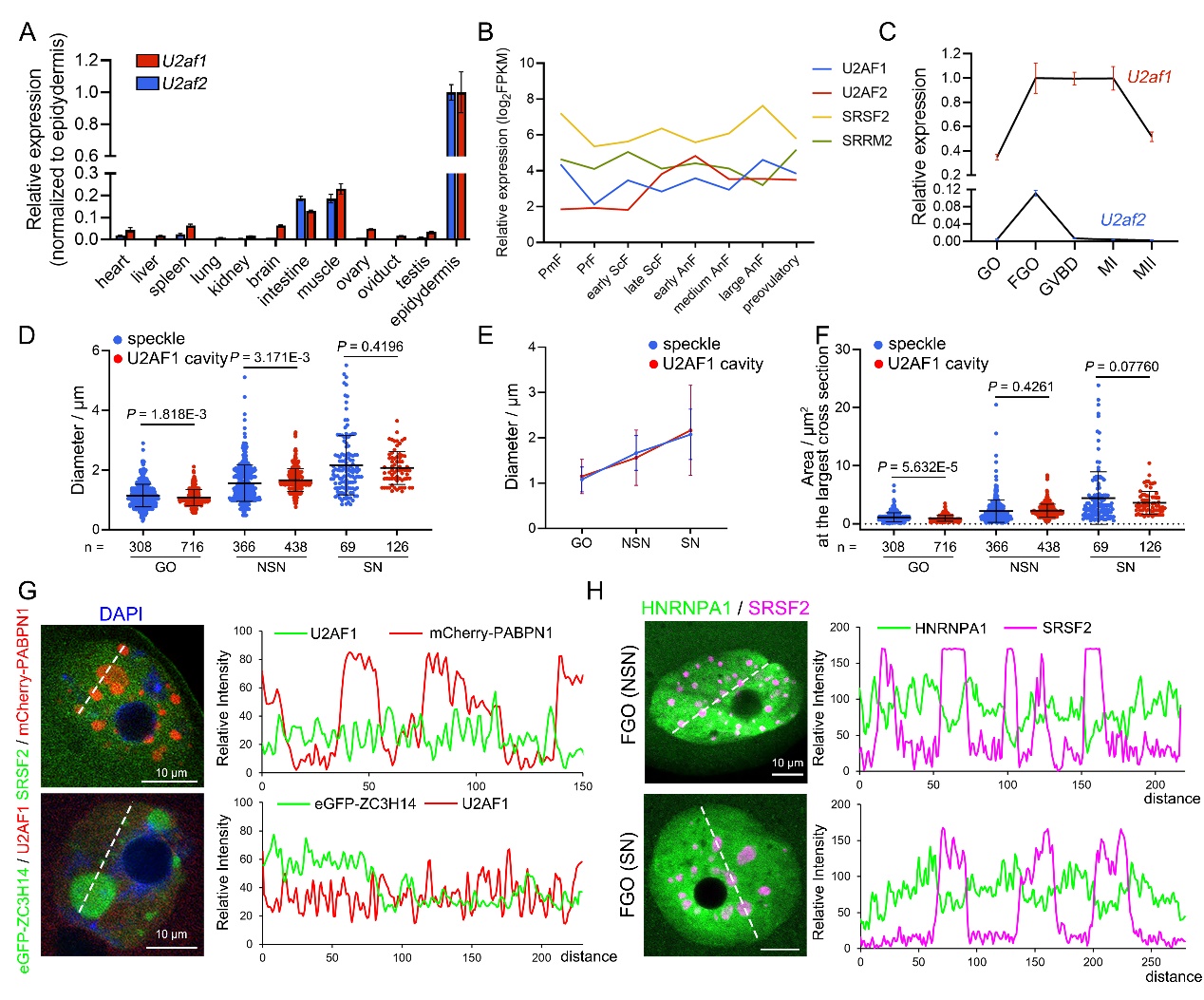


**Supplementary Figure.2 U2AF1 forms cavities and distributes exclusively outside nuclear speckles in mouse oocytes. A:** qRT-PCR results showing the relative expression levels of *U2af1/2* transcripts in mouse tissues. Mean ± SEM. n = 3. **B:** Relative transcript abundance in oocytes at the indicated follicle stage, referred to Liu W., *et al* (2023). PmF, primordial follicle. PrF, primary follicle. ScF, secondary follicle. AnF, antral follicle. **C:** qRT-PCR results showing the relative expression levels of *U2af1/2* transcripts in mouse oocytes during meiotic maturation. Mean ± SEM. n = 3. **D-E:** Comparison of the diameters of nuclear speckles and U2AF1 cavities, corresponding to Figure. 3. Mean ± SEM. The numbers of the analyzed speckles and cavities (n) are annotated at the bottom. **F:** Comparison of the cross-section areas of the diameters of nuclear speckles and U2AF1 cavities. Mean ± SEM. The numbers of the analyzed speckles and cavities (n) are annotated at the bottom. **G-H:** Comparison of the localization of U2AF1 and ectopically expressed PABPN1, and ZC3H14 (**G**), and endogenous U2AF1 and HNRNPA1 (**H**) in mouse oocytes. The distribution of the indicated proteins was analyzed along the white dashed lines using ImageJ. Scale bars, 10 μm.


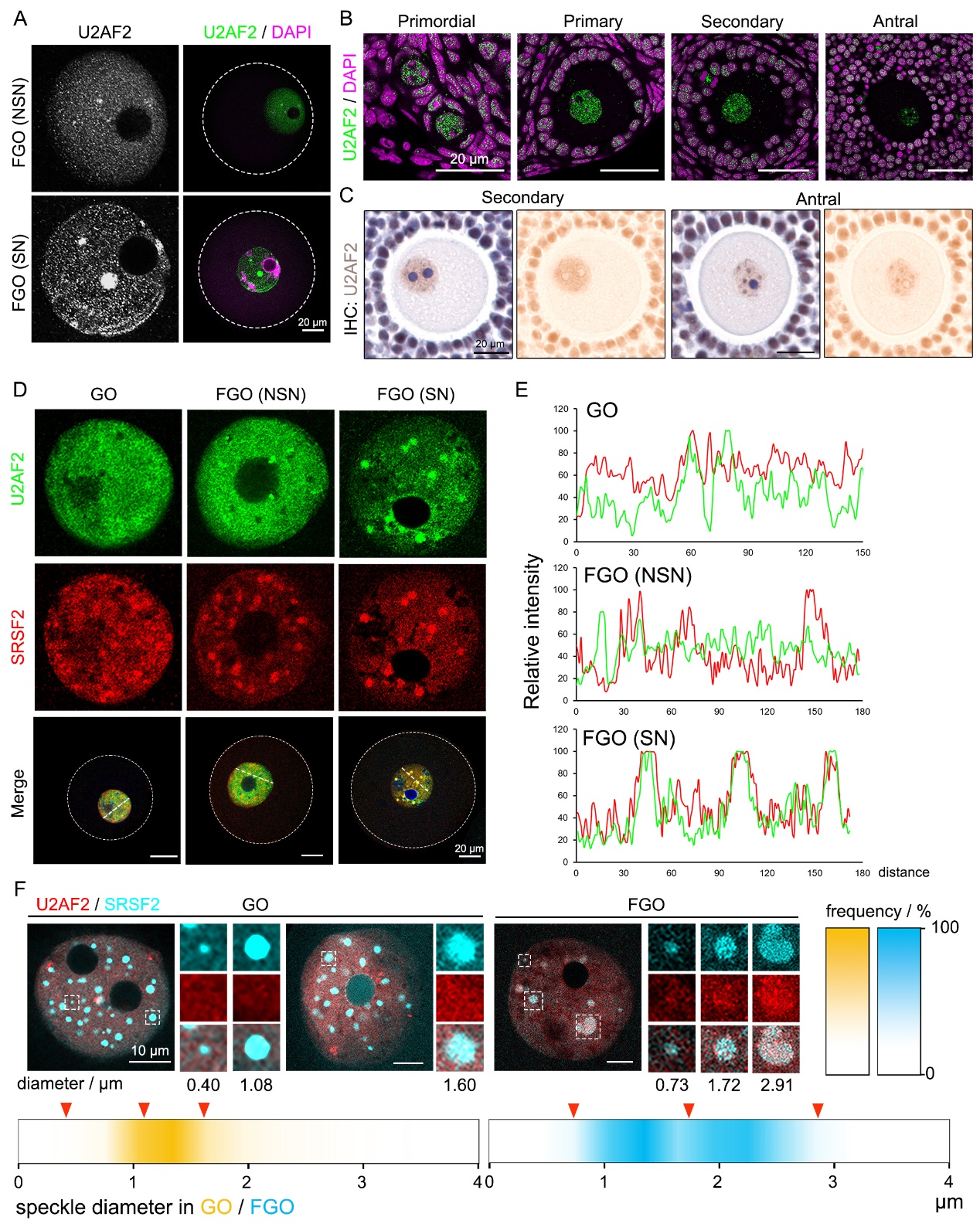


**Supplementary Figure. 3 U2AF2 serves as a nucleoplasmic protein in mouse oocytes and assemblies into the nuclear speckles only at SN stage. A-B:** IF staining of U2AF1 in FGOs (**A**) and the indicated follicle stages (**B**). Scale bars, 20 μm. **C:** IHC staining of U2AF2 in secondary and antral follicles. U2AF2 signals were separated using ImageJ. Scale bars, 20 μm. **D-E:** IF-staining of U2AF2 and SRSF2 in GOs and FGOs. The distribution of U2AF2 and SRSF2 (**E**) was analyzed along the white dashed lines using ImageJ. Scale bars, 20 μm. **F:** IF results showing U2AF2 distribution without/within representative nuclear speckles in mouse oocytes (top). The frequencies of speckle diameters are indicated by colors from white to yellow and blue, in GOs and FGOs, respectively (bottom). The red triangles indicate the speckle diameters in enlarged views. Scale bars, 10 μm.


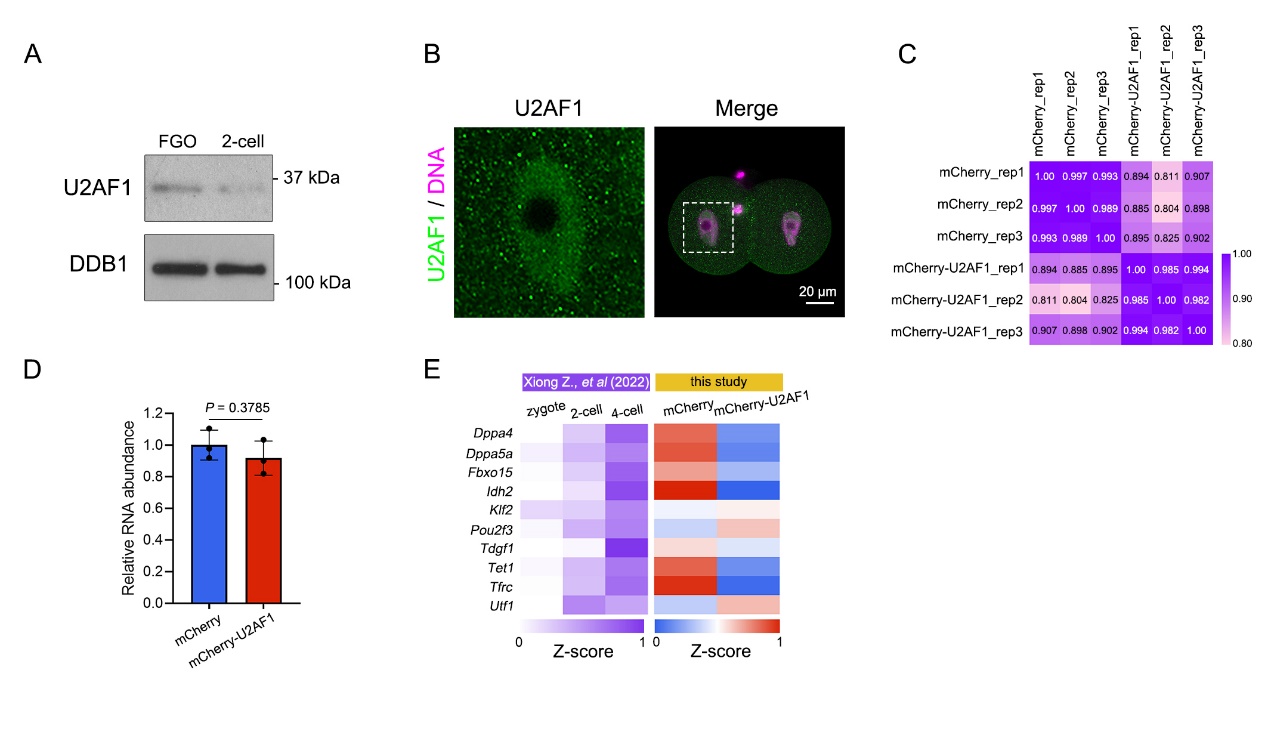


**Supplementary Figure. 4 U2AF1 expresses in mouse 2-cell embryos, and ectopically expressed U2AF1 altered 2-cell-staged transcriptome. A:** Western blotting results showing U2AF1 expression in FGOs and 2-cell embryos. DDB1 served as the loading control. **B:** IF staining of U2AF1 in the 2-cell embryo. Scale bars, 20 μm. **C:** Correlation among the three replicate measurements using the FPKM for each transcript of the indicated groups. The correlation coefficient was calculated by Pearson’s correlation. **D:** Relative RNA abundance of the indicated groups analyzed through ERCC incorporation. Mean ± SD. **E:** Heatmaps presenting the expression of representative pluoripotent gene transcripts in WT embryos (left, referred to Xiong Z., *et al.*) and U2AF1 hyperexpression 2-cell embryos (this study, right).


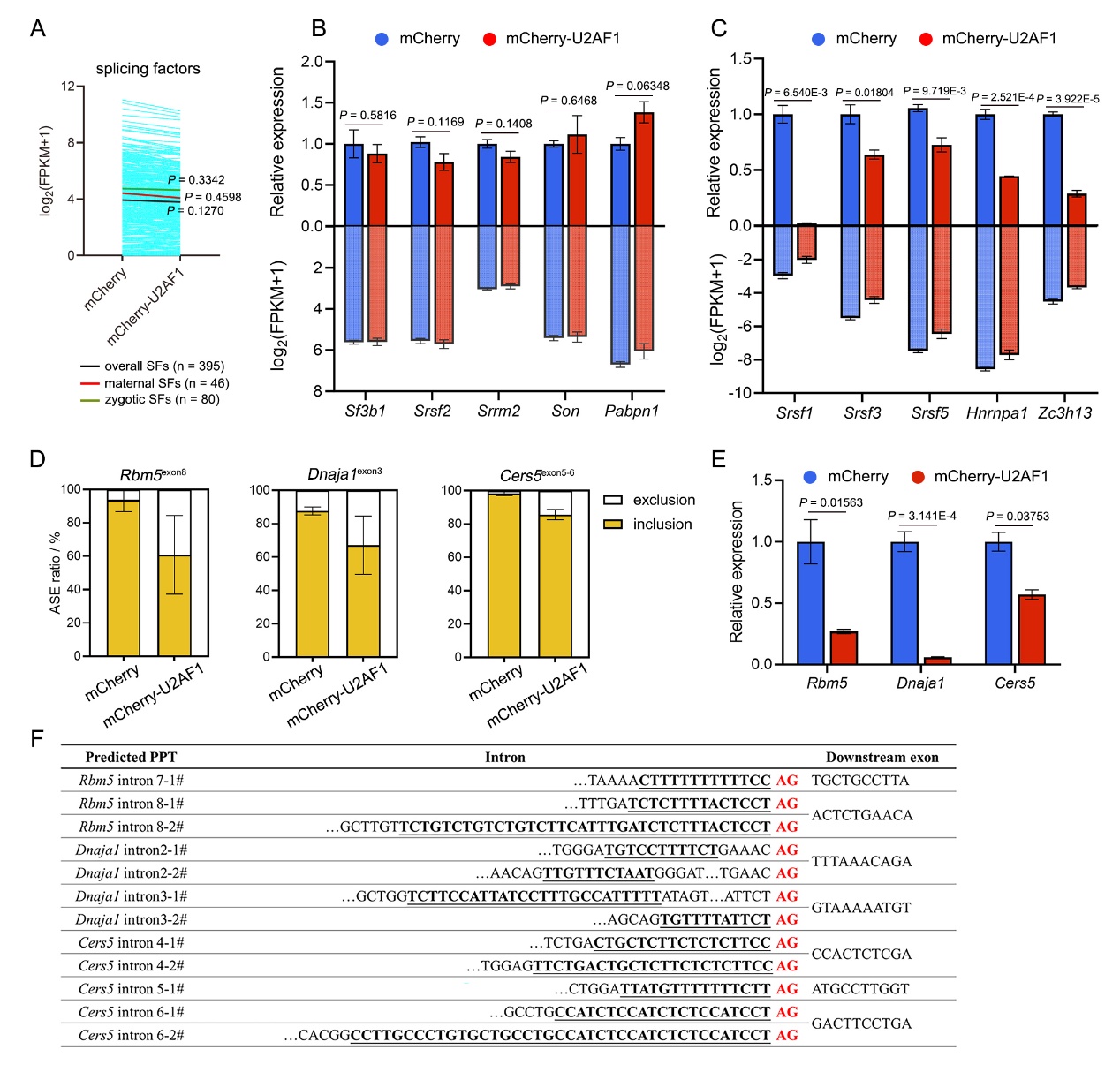


**Supplementary Figure. 5 Excessive U2AF1 resulted in exon usage and transcript isoform alternations. A:** Expression of splicing factors, including maternal (red) and zygotic (green) ones detected in the transcriptomes. SF, splicing factor. **B-C:** Expression abundance of representative SFs and nuclear speckle components detected by qRT-PCR analyses and in the transcriptomes. Transcripts either maintained (**B**) or downregulated (**C**) were presented. Mean ± SEM. n = 3. **D:** Bar charts presenting the inclusion levels of the indicated exons in U2AF1-hyperexpressing 2-cell embryos, corresponding to Figure. 5D-E. Mean ± SD. n = 3. **E:** qRT-PCR results showing the abundance of the indicated mRNA isoforms. Mean ± SEM. n = 3. **F:** Predicted PPTs upstream the *Rbm5*^exon7^, *Dnaja1*^exon2^, and *Cers5*^exon5/6^ cassettes, according to the complementary DNA database. The PPTs are underlined and in bold fonts. AG dinucleotides at the 3'-splice sites are indicated in red.


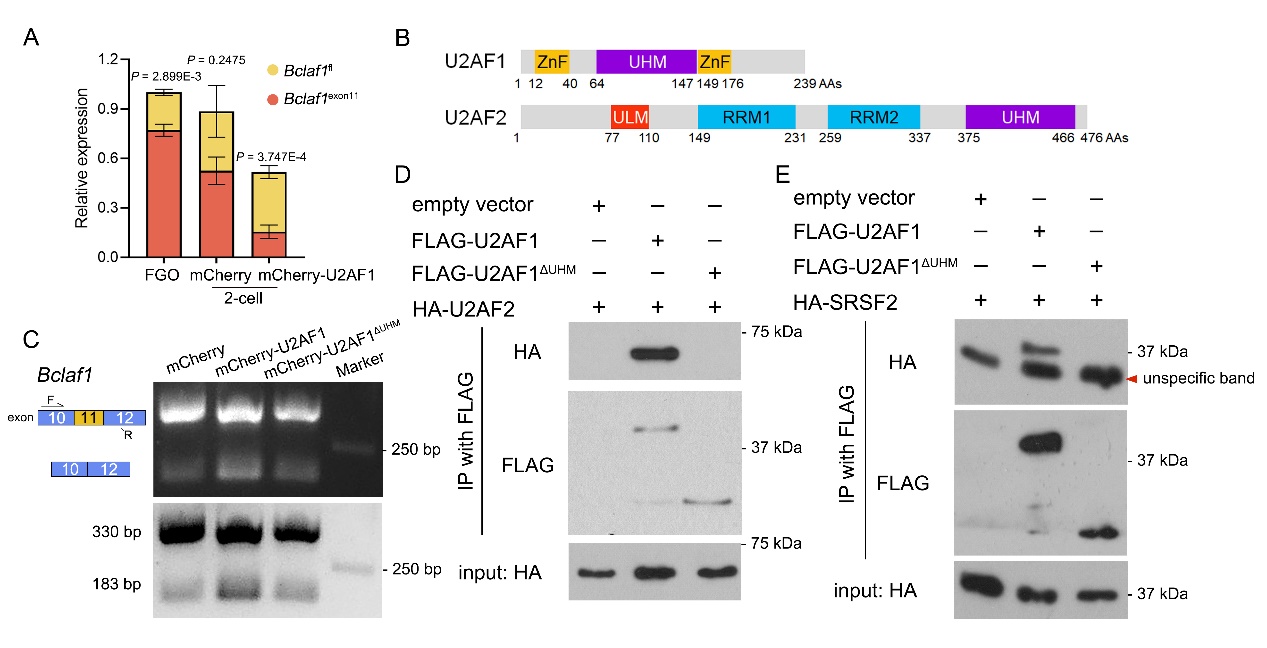


**Supplementary Figure. 6 U2AF1 hyperexpression results in *Bclaf1*^exon11^ exclusion and tunes BCLAF1 distribution in oocytes. A:** qRT-PCR results showing *Bclaf1*^exon11^ and *Bclaf1* transcripts in FGOs, mCherry- and mCherry-U2AF1-expressing 2-cell embryos. Mean ± SEM. n = 3. The relative expression levels are presented in overlaid bar charts, not stacked ones, with *Bclaf1*^exon11^ and *Bclaf1*^fl^ using the same axis. **B:** Schematic illustration of U2AF1/2 protein and their domains. ZnF, zinc finger. UHM, U2AF homology motif. ULM, U2AF ligand motif. RRM, RNA-recognizing motif. **C:** PCR analyses of *Bclaf1* mRNA variants in mCherry, mCherry-U2AF1, and mCherry-U2AF1^ΔUHM^-expressing FGOs. **D-E:** Co-immunoprecipitation (co-IP) results showing the interactions between HA-tagged U2AF2 (**F**) and SRSF2 (**G**), and FLAG-tagged U2AF1 isoforms.


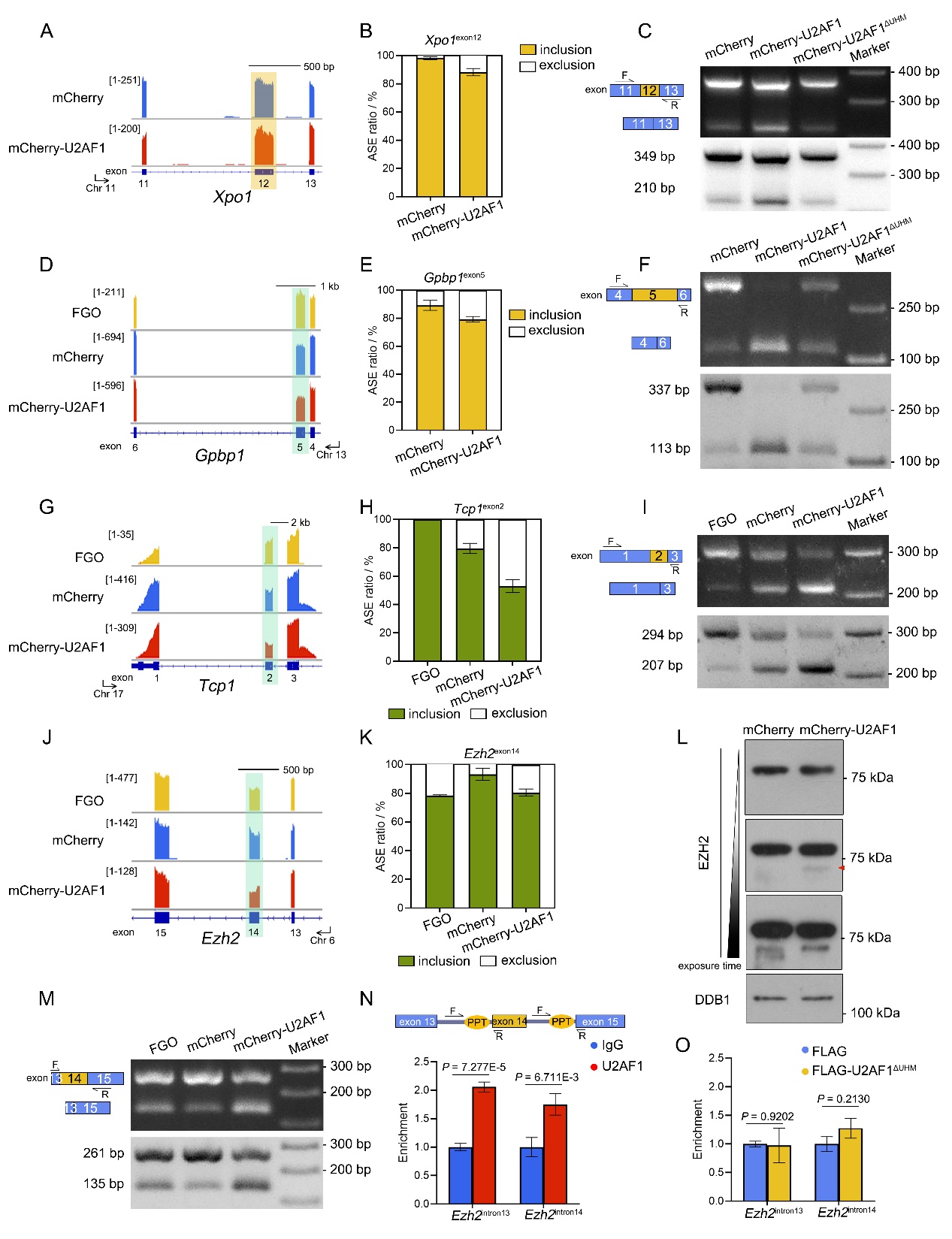


**Supplementary Figure. 7 U2AF1 drove massive exon exclusion in 2-cell embryos. A-C:** IGV illustration, bar chart and PCR analysis presenting *Xpo1* transcripts in the U2AF1 ectopically expressed 2-cell embryos and their cohorts. **D-E:** IGV illustration and bar chart showing *Gpbp1* transcripts. **F:** PCR analysis of *Gpbp1* variants in 2-cells ectopically expressing mCherry, mCherry-U2AF1, and mCherry-U2AF1^ΔUHM^, respectively. **G-I:** IGV illustration, bar chart, and PCR analysis of *Tcp1* variants in FGOs, and 2-cells expressing the indicated vectors. **J-K:** IGV illustration and bar chart presenting *Ezh2*^exon14^-carrying variants in FGOs and 2-cell embryos. **L:** Western blotting results of EZH2 isoforms in U2AF1-hyperexpressing 2-cells. Red triangle points at the exon14-lacking isoform. DDB1 served as the loading control. **M:** PCR analysis of *Ezh2* variants in FGOs and 2-cell embryos. **N-O:** qRT-PCR results showing endogenous U2AF1 (**N**) and U2AF1^ΔUHM^ (**O**) affinity to different regions within *Ezh2* transcripts detected by RIP. Mean ± SEM. n = 3. The design of primers is annotated on the top of **N**.
